# A novel approach to analyze lysosomal dysfunctions through subcellular proteomics and lipidomics: the case of NPC1 deficiency

**DOI:** 10.1038/srep41408

**Published:** 2017-01-30

**Authors:** Arun Kumar Tharkeshwar, Jesse Trekker, Wendy Vermeire, Jarne Pauwels, Ragna Sannerud, David A. Priestman, Danielle te Vruchte, Katlijn Vints, Pieter Baatsen, Jean-Paul Decuypere, Huiqi Lu, Shaun Martin, Peter Vangheluwe, Johannes V. Swinnen, Liesbet Lagae, Francis Impens, Frances M. Platt, Kris Gevaert, Wim Annaert

**Affiliations:** 1 Laboratory for Membrane Trafficking, VIB Center for Brain & Disease Research, KU Leuven, Department of Neurosciences, Leuven, Belgium; 2 Department of Life Science Technology, imec, Leuven, Belgium; 3 VIB Medical Biotechnology Center & Department of Biochemistry, UGent, Ghent, Belgium; 4 Biomedical MRI, Department of Imaging and Pathology, KU Leuven, Leuven 3000, Belgium; 5 Department of Pharmacology, University of Oxford, Oxford, United Kingdom; 6 VIB Bio-imaging core VIB Center for Brain & Disease Research, Leuven, Belgium; 7 Laboratory of Cellular Transport Systems, Department of Cellular and Molecular Medicine, KU Leuven, Leuven, Belgium; 8 Laboratory of Lipid Metabolism and Cancer, Department of Oncology, KU Leuven, Leuven, Belgium; 9 Department of Physics, Solid State Physics and Magnetism, KU Leuven, Leuven, Belgium; 10 Proteomics Expertise Center, VIB Medical Biotechnology Center, Ghent, Belgium

## Abstract

Superparamagnetic iron oxide nanoparticles (SPIONs) have mainly been used as cellular carriers for genes and therapeutic products, while their use in subcellular organelle isolation remains underexploited. We engineered SPIONs targeting distinct subcellular compartments. Dimercaptosuccinic acid-coated SPIONs are internalized and accumulate in late endosomes/lysosomes, while aminolipid-SPIONs reside at the plasma membrane. These features allowed us to establish standardized magnetic isolation procedures for these membrane compartments with a yield and purity permitting proteomic and lipidomic profiling. We validated our approach by comparing the biomolecular compositions of lysosomes and plasma membranes isolated from wild-type and Niemann-Pick disease type C1 (NPC1) deficient cells. While the accumulation of cholesterol and glycosphingolipids is seen as a primary hallmark of NPC1 deficiency, our lipidomics analysis revealed the buildup of several species of glycerophospholipids and other storage lipids in selectively late endosomes/lysosomes of NPC1-KO cells. While the plasma membrane proteome remained largely invariable, we observed pronounced alterations in several proteins linked to autophagy and lysosomal catabolism reflecting vesicular transport obstruction and defective lysosomal turnover resulting from NPC1 deficiency. Thus the use of SPIONs provides a major advancement in fingerprinting subcellular compartments, with an increased potential to identify disease-related alterations in their biomolecular compositions.

Human cells may well express over 10,000 different proteins^1^,^2^ and 1,000 different lipid species^3^,^4^, that are spatially distributed into distinct subcellular compartments^5^,^6^,^7^. To carry out their physiological function, these biomolecules must be appropriately directed to and remain present at their correct subcellular location, or are locally modified to mediate trafficking or signaling regulation^8^. The ability of proteins and lipids to associate with resident subcellular compartments provides crucial information about their local activity, interacting molecules and functions. When linked to human diseases, such information may contribute to our understanding of the underlying pathogenesis^9^,^10^,^11^.

Proteomics and lipidomics are used as unbiased approaches to explore the molecular basis of disease^6^,^12^,^13^,^14^,^15^. It enables identifying affected complex metabolic and signaling pathways to better understand pathoetiology^16^,^17^,^18^. Despite major advances in mass spectrometry, the enormous protein and lipid complexities remain a challenge to resolve. This complexity can be significantly reduced through the analysis of purified subcellular compartments, as it enhances the detection of low abundant components^19^,^20^. A major caveat is the purity of the organelles, as presence of minute contaminants may hinder identification or may incorrectly link identified proteins to organelles^6^,^19^,^21^. Most reliable omics data have been obtained for specific organelle structures, like mitochondria, synaptic vesicles, and centrosomes, which, because of their homogeneity and biophysical features, allow for high yield and pure isolation^22^,^23^,^24^. The dynamic nature and overlapping physicochemical parameters make other organelles such as the plasma membrane (PM) and endosomal/lysosomal compartments difficult to isolate^9^. To solve this, we developed a magnetic isolation strategy using superparamagnetic iron oxide nanoparticles (SPIONs).

SPIONs with a large surface area to volume ratio and superparamagnetic properties hold great promise for their applications in biology and medicine^25^,^26^,^27^. In general, SPIONs have an inorganic magnetic core and an organic/inorganic shell providing stability, charge and overall chemical functionality. Thus far, they were mostly used for drug delivery, hyperthermia treatment and contrast enhancement in magnetic resonance imaging (MRI)^28^,^29^. However, for targeting subcellular compartments, their efficacy is highly dependent on size, shape, magnetic and surface properties^30^,^31^,^32^,^33^ proving the need for specific synthesis methods^34^. Previous attempts using nanoparticles such as colloidal iron coated with high-molecular weight dextran to magnetically isolate lysosomes (LYS) from amoeba^35^ and mammalian cells^36^,^37^ led to lower yields due to the unstable nature of nanoparticles and their increased cellular toxicity. This might have been related to the synthesis method based on co-precipitation of iron salts in aqueous alkaline solutions in the presence of stabilizers^38^,^39^,^40^, which fails to properly control average particle size, size distribution, surface functionalization^41^, thereby increasing cellular toxicity. While this approach may not be appropriate for purifying LYS, they provided an alternative to density gradient centrifugation-based enrichments of such organelles.

Here, we used thermal decomposition and ligand-exchange or ligand-addition to prepare SPIONs with tailor-made surface properties for the assessment of distinct nanoparticle-cell interactions. We found that aminolipid-coated SPIONs remained adhered to the PM, whereas dimercaptosuccinic acid (DMSA)-coated SPIONs were efficiently targeted to late endosomes(LE)/LYS. This enabled the development of a standardized isolation methodology that we validated by omics profiling of PMs and LE/LYS isolated from wild-type and Niemann-Pick type C1 (NPC1)-deficient cells.

Niemann-Pick disease type C (NPC) is an inherited severe lysosomal storage disorder, which in 95% of affected individuals is caused by loss of function of NPC1^42^,^43^. NPC1 is a multi-spanning membrane protein localized primarily in the limiting membrane of LE/LYS^44^,^45^,^46^ where together with NPC2 it regulates cholesterol export from LYS^47^,^48^,^49^. A mutation in either protein results in the accumulation of lipids including cholesterol (ST), glycosphingolipids (GSL) and sphingomyelin (SM) in LE/LYS^50^,^51^. We therefore investigated the effects of NPC1 deficiency (KO) on the biomolecular composition of PMs and LE/LYS to better understand the cellular pathophysiology of this disease. To our surprise and despite severe aberrant endo-lysosomal transport, the proteome and lipidome of isolated PMs of NPC1-KO cells remain largely unaltered. In contrast, the lipid composition of isolated LE/LYS dramatically changed together with specific changes in their protein composition. Thus, omics analysis of magnetically isolated PMs and LE/LYS allows for the first time the spatial identification of quantitative and qualitative alterations in their biomolecular composition at much higher resolution and sensitivity than was previously achieved. This method provides a powerful toolbox in the context of disease analysis, including lysosomal storage diseases, but potentially extended to other neurodegenerative diseases involving endo/lysosomal transport defects.

## Results

### Synthesis and characterization of SPIONs

**Figure 1:**
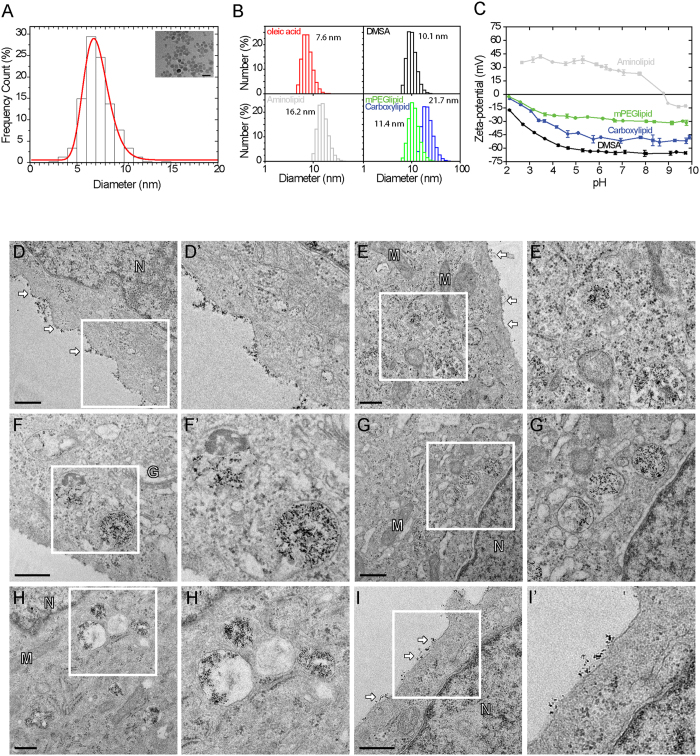
DMSA-SPIONs are internalized whereas amino lipid-SPIONs remain on the surface. Physical and chemical characterization using TEM to measure core size **(A)**, DLS to measure hydrodynamic size **(B)**, and zeta-potential **(C)**. TEM images and zoom (corresponding white square) of HeLa cells incubated with DMSA- **(D** to **H)** or amino lipid-SPIONs **(I)** for 15 min at 37 °C. Different chase periods were used for DMSA-SPIONs: **(D)** 0 min, **(E)** 1 h, **(F)** 2 h, **(G)** 3 h, **(H)** 4 h. A chase period of 2 h was used for the aminolipid-SPIONs **(I)**. Scale bar is 0.5 μm.

SPIONs synthesized using high-temperature organic phase decomposition of iron precursors (named thermal decomposition, Supp. Fig. S1A) are monocrystalline, spherical and have a narrow size distribution (9 nm ± 0.02) (Fig. 1A). Their superparamagnetic behavior was confirmed by vibrating sample magnetometry (VSM) showing no remnant magnetization at zero applied field (Supp. Fig. S1B,C).

To render them water-dispersible, SPIONs are functionalized. Firstly, by ligand addition, the hydrophobic tail of PEGylated phospholipids interacts with the oleic acid coating of SPIONs through van der Waals interactions. The PEG chain and the hydrophilic head groups (amino/carboxyl/methoxy) make these SPIONs water-dispersible^52^. Alternatively, the oleic acid coat was substituted with DMSA. Herein surface functionalization is based on chemisorption of carboxylate groups to the iron oxide surface and disulfide linkage between the thiol groups in DMSA^53^. Functionalized SPIONs exhibited excellent water dispersibility, retained narrow size distribution and core size (Supp. Fig. S1H–M). Magnetic hysteresis measurements confirmed that magnetic behavior was reversible (Supp. Fig. S1D–G) as expected for particles <15 nm^54^ and no changes related to the surface coating were observed, implying that functionalization did not affect magnetic properties. Dynamic light scattering (DLS) showed a small increase in hydrodynamic diameters of the functionalized SPIONs (Fig. 1B) versus transmission electron microscopy (TEM) (Supp. Fig. S1I–L) consistent with literature^55^,^56^,^57^. Further, FTIR confirmed the introduction of functionalized surface coatings (for details, see Supp. Fig. S1N). Subsequent zeta- potential measurements demonstrated that amino end group SPIONs are cationic between pH 2–8 and anionic between pH 9–10, with an isoelectric point of 9 while methoxy-, carboxy- and DMSA- SPIONs were anionic throughout all pH ranges (Fig. 1C).

### Nanoparticle uptake and isolation of subcellular compartments

We incubated HeLa cells for 15 min at 37 °C with different functionalized SPIONs and subsequently chased in fresh medium for varying time periods (0-to-4 h). DMSA-SPIONs behaved as expected for solutes to be taken up by cells. From a preferred presence at the cell surface during the pulse (Fig. 1D,D’) they gradually become depleted from the PM and appeared in successive endosomal compartments (Fig. 1E–H’). At the longest chase (3–4 h), DMSA-SPIONs were confined to electron-dense rounded organelles reminiscent of LE/LYS (Supp. Fig. 3A). Surprisingly, aminolipid-SPIONs remained adhered to the cell surface even after extended chase periods (Fig. 1I,I’ and Supp. Fig. 3B). Carboxyl- and methoxy-SPIONs failed to become internalized and were abandoned (Supp. Fig. 2B,C).

Importantly, DMSA- and aminolipid-coated SPIONs did not induce alterations in BiP/Grp78 levels nor in the spliced form of X-box protein 1 (XBP1) indicating that these SPIONs do not cause ER stress (Supp. Fig. 2D–F). Functionalized SPIONs did not significantly affect cell viability as measured by trypan blue staining (Supp. Fig. 2A) nor did they activate cell death pathways (Supp. Fig. 2G,H). DMSA-SPIONs neither affected internalization or early endocytic events as determined by transferrin receptor internalization (Supp. Fig. 2I and L,M). Finally, DQ-BSA assays revealed no differences in lysosomal proteolytic activity in cells treated with or without DMSA-SPIONs (Supp. Fig. 2J).

**Figure 2:**
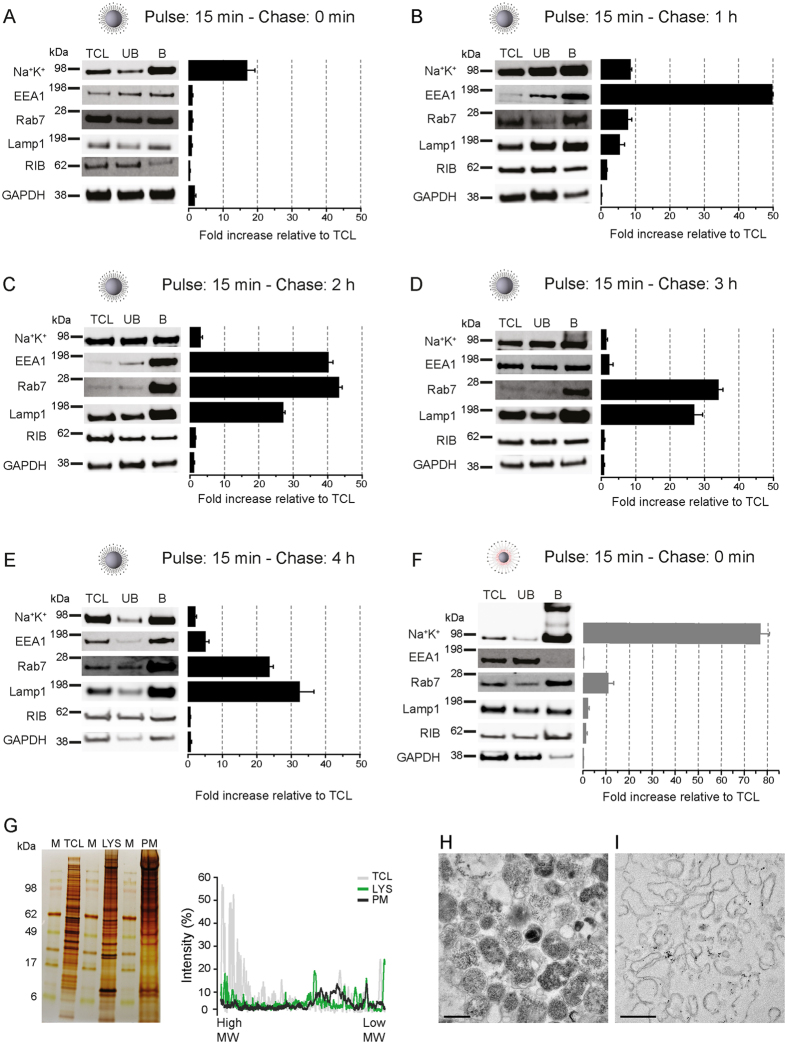
DMSA-SPIONs accumulate in endosomal compartments with increasing chase periods while aminolipid-SPIONs remain tethered at the cell surface. (**A**) Quantitative immunoblot analysis (equal protein loading) for the indicated organelle marker proteins in isolated fractions using DMSA-SPIONs with increasing chase periods of (**A**) 0 min, (**B**) 1 h, (**C**) 2 h, (**D**) 3 h, (**E**) 4 h or in fractions isolated using aminolipid-SPIONs (**F**) as a fold increase relative to total cell lysate (mean ± SEM, n = 3). Na^+^K^+^ (Na^+^K^+^-ATPase) is a PM-localized integral membrane protein, EEA1 marks early endosomes, Rab7 late endosomes and Lamp1 lysosomes. RIB (Ribophorin) and GAPDH represent endoplasmic reticulum and cytosol, respectively. TCL - total cell lysate; UB - Unbound/non-magnetic fraction and B - Bound/magnetic fraction. (**G**) Silver staining of total cell lysate (TCL), bound/magnetic fraction isolated using SPIONs functionalized with DMSA (LYS) or with aminolipids (PM). The distinct protein profile in the bound fraction as observed by the lane scan underscores the enrichment of specific protein subsets (M - Marker, SeeBlue plus2 rainbow protein marker (Invitrogen)). TEM analysis of the fractions isolated using DMSA- (**H**) and aminolipid- (**I**) coated SPIONs. Scale bar = 0.5 μm.

We next exploited the distinct features of DMSA- versus aminolipid-SPIONs for the magnetic isolation of LE/LYS and PMs, respectively. Since aminolipid-SPIONs remained at the cell surface irrespective of the chase period we restricted the protocol to 15 min incubation at 4 °C for aminolipid-SPIONs. Following the 4 °C incubation of aminolipid-SPIONs and the different chase periods for DMSA-SPIONs (37 °C), SPION-loaded compartments were magnetically isolated from the post-nuclear supernatant. Subsequent immunoblot analysis was largely in agreement with TEM (Fig. 1D–I’). A 15 min pulse resulted in the enrichment of PM markers like Na^+^K^+^-ATPase in both DMSA- and aminolipid-SPION isolates (Fig. 2A,F). For DMSA-SPION isolations, Na^+^K^+^-ATPase immunoreactivity declined gradually with increasing chase-time indicating efficient internalization. In agreement with transit through endosomes, DMSA-SPIONs maximally co-enriched with early endosomal antigen 1 protein (EEA1) and Rab5 after 1 h (Fig. 2B), and with LE Rab7 and LAMP-1 after 2 h (Fig. 2C) and beyond (Supp. Fig. 4). Longer chase periods did not result in further enrichment of LE/LYS markers indicating saturation of DMSA-SPIONs in late compartments (Fig. 2D,E). The lack of enrichment (<1 fold) of mitochondria, ER, peroxisomes and cytoskeletal elements underscore the very low levels of contamination and high purities of isolated LE/LYS (Supp. Fig. 4) which was confirmed by SDS-PAGE and silverstaining (Fig. 2G). Distinct and specific protein subsets were enriched in LE/LYS and PM as compared to the total cell lysate (TCL).

TEM analysis demonstrated that DMSA-SPIONs internalized for 4 h were found abundantly concentrated in electron-dense organelles often with a multilamellar appearance or the presence of intraluminal vesicles (Fig. 2H) characteristic for LYS and LE/multivesicular bodies (MVBs), respectively. Further isolated LE/LYS remain intact with a high latency of 67% ± 0.03 (n = 3) as measured by β-hexosaminidase activity^37^ and could be still stained with Lysotracker indicating their acidic nature (Supp. Fig. 2K). In contrast, aminolipid-SPION isolates comprised long membranous sheets, decorated with nanoparticles reminiscent of PMs (Fig. 2I). In agreement with immunoblots, SPION-isolated compartments were virtually devoid of other contaminating organelles underscoring their high purity. By avoiding time-consuming fractionation procedures, high yields (on average 70–120 μg and 80–120 μg of protein for purified PMs and LE/LYS, respectively) were achieved permitting subsequent omics analysis.

### Proteome analysis of isolated compartments

We next performed proteome analysis on purified PMs and LE/LYS using gel-based protein separation prior to MS analysis (see M&M). Considering those proteins identified in replicate experiments and by at least two peptides, we identified 3,100, 1,046, 3,196 proteins in TCL, PM and LYS fractions, respectively (Supp. Fig. 5A1–A3).

DAVID Gene Ontology (GO) tool^58^ was used to assess the enrichment of specific protein groups versus TCL. GO Functional Annotation tool was used to list top terms (i.e., with a minimum 2-fold enrichment and p-value ≤ 0.05) enriched in LYS (Supp. Fig. 5B1,B2) and PM (Supp. Fig. 5C1,C2) fractions relative to TCL. In LYS, 36% of identified proteins (1136/3196) are significantly annotated as ‘membrane-enclosed lumen’ (p-value: 2.6E-130) followed by GO terms including ‘envelope’, ‘organelle membrane’ and ‘endomembrane system’ (p-values: 1.30E-60, 2.50E-45 and 1.40E-19, respectively). The PM fraction is highly represented (over 40% of all identified proteins; p-value: 1.40E-32) by authentic membrane proteins and annotated with GO terms underscoring a highly purified fraction (‘integral/intrinsic to membrane’, ‘plasma membrane’). As expected, annotations to contaminating organelles like mitochondria, ER, Golgi and nucleus are very low to scarce, underscoring the high purity and confirming previous biochemical/ultrastructural analysis (Fig. 2). It should be noted that protein interpretation in LE/LYS is complicated by the fact that non-lysosomal constituents in these samples may as well represent proteins that are destined for degradation (protein catabolism) and that were thus delivered to lysosomes from other compartments like LE and autophagosomes. As PMs and LE/LYS isolates are compatible with downstream proteomic analysis we next applied this in a disease context by comparing PMs and LE/LYS isolated from wild-type and NPC1-KO cells.

### Generation of NPC1-KO cells

NPC1 was knocked out in HeLa cells using CRISPR/Cas9 genome editing. Two differently targeted and independent KO clones were selected through subcloning. Western blot confirmed complete absence of NPC1 protein in both clones (Supp. Fig. 6A), while filipin staining revealed an excessive intracellular accumulation of cholesterol in LAMP1-positive organelles (Supp. Fig. 6D). A similar phenotype is observed when HeLa cells are treated with U18666A, an inhibitor of NPC1 function^59^ widely used to pharmacologically induce NPC disease (Supp. Fig. 6D). Moreover, NPC1-KO cells presented with a relative increase in the volume of acidic compartments (Supp. Fig. 6B). LysoSensor DND189 showed an apparent drop in luminal pH (Supp. Fig. 6C) which was not observed in NPC1-KO CHO cells^51^ and requires further investigation. On the other hand, increase in sphingosine (Supp. Fig. 6E) and GSL levels (Supp. Fig. 6F) is reminiscent of NPC1-KO CHO cells^51^,^60^. Subtle differences between both NPC1-KO clones may originate from clonal selection generating clones with slightly different genetic backgrounds.

### SPION-based isolation of PMs and LE/LYS

**Figure 3:**
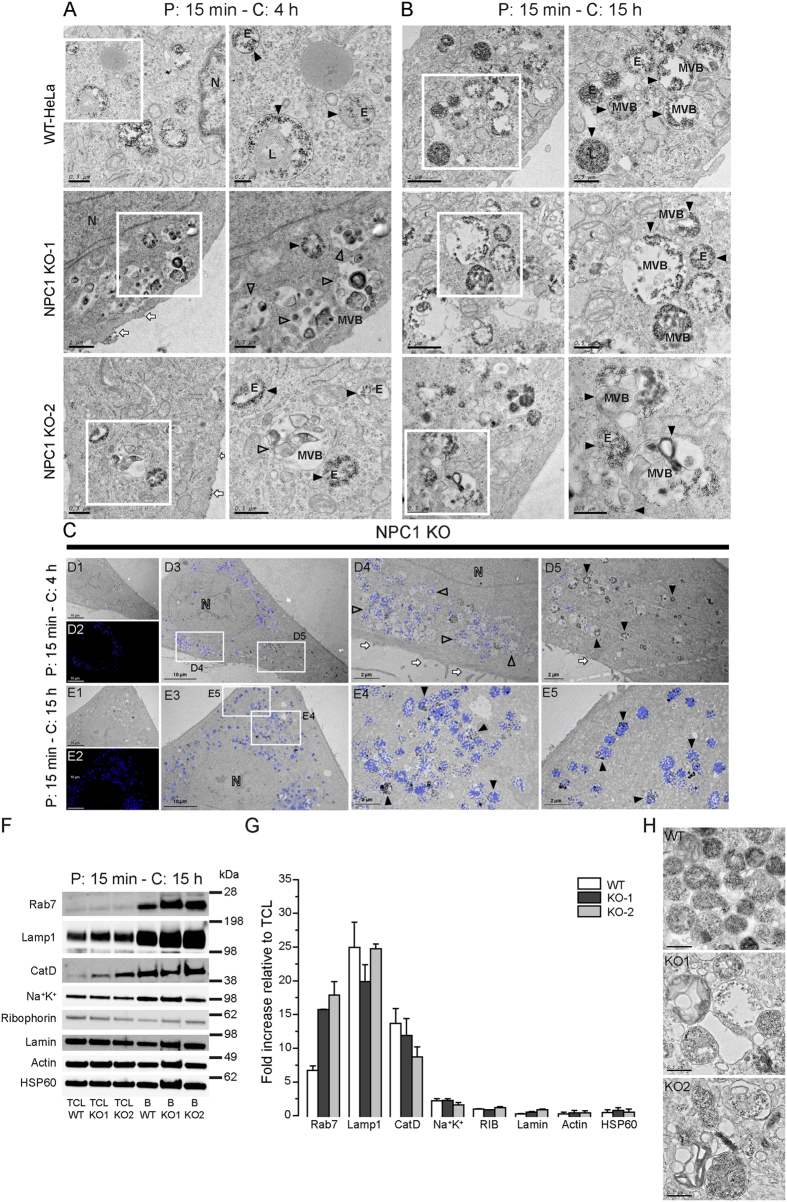
Functional loss of NPC1 delays late events in the lysosomal degradation pathway. TEM images and zoom (corresponding white square) of WT and NPC1-KO HeLa cells incubated with DMSA-SPIONs for 4 h **(A)** and 15 h **(B)**. Indicated are LE/LYS containing SPIONs (filled arrow heads), as well as devoid of SPIONs (empty arrow heads). **(C)** SIM-CLEM analysis of NPC1-KO cells after a chase of 4 h showing filipin-positive organelles devoid of SPIONs (empty arrow heads). An increase in chase time to 15 h results in abundant co-localization of SPIONs with filipin-positive degradative organelles. (**D1-3** and **E1-3**) TEM and SIM-overview: areas of interest (corresponding white squares) were analyzed by CLEM (**D4-5** and **E4-5**). N: nucleus, E: endosomes, L: lysosomes, MVB: multi-vesicular bodies. Scale bar = 10 μm (**D1-3** and **E1-3**) and 2 μm (**D4-5** and **E4-5**). Quantitative Western blot analysis **(F**,**G)** of the indicated organelle marker proteins in fractions isolated using DMSA-SPIONs (15 h chase) expressed as a fold increase relative to total cell lysate (TCL) (mean ± SEM, n = 3). Na^+^K^+^ (Na^+^K^+^-ATPase) is used as marker for PM, Rab7 represents late endosomes, Lamp1, CatD (Cathepsin D) lysosomes and Lamin is a nuclear marker. HSP60, ribophorin and actin represent mitochondria, ER and cytoskeleton, respectively. TCL - total cell lysate and B - Bound/magnetic fraction. TEM analysis of the fractions isolated from WT, NPC1-KO1 and NPC1-KO2 HeLa cells using DMSA-SPIONs following a chase period of 15 h **(H)**. Scale bar = 0.5 μm.

We next used aminolipid- and DMSA-SPIONs to isolate PMs and LE/LYS from wild-type and NPC1-KO HeLa cells. TEM analysis revealed that after 4 h, in NPC1-KO cells, DMSA-SPIONs localized to electron-dense organelles (Fig. 3A) resembling LYS compartments (filled arrowheads), much like the case in WT cells. A second major perinuclear population of highly proliferated vesicular organelles, including multi-lamellar structures, were devoid of SPIONs (empty arrowheads). Using correlative structured illumination microscopy and EM (SIM-CLEM), these degradative organelles were filipin-positive, but lacked SPIONs (Fig. 3C, empty arrowheads in D4), while SPION-filled lysosomal structures were largely filipin-negative (Fig. 3C, filled arrowheads in D5). These data underscore a significant delay in transport to/fusion with LE/LYS as compared to wild-type cells, further supported by a longer latency of DMSA-SPIONs at the surface of NPC1-KO cells (Fig. 3A, white arrows). In agreement, DMSA-SPIONs reached these pleiomorphic, filipin-positive LE/LYS-like structures when increasing the chase time from 4 to 15 h (Fig. 3B,C, panel E4-E5, filled arrowheads).

This optimized chase time was used to magnetically isolate LE/LYS from WT and NPC1-KO cells, while the PM protocol remained unaltered. Consistent with previous data, Western blotting of isolated LE/LYS showed a strong co-enrichment with Rab7 and LAMP1 in both WT and NPC1-KO cells while marker proteins from contaminating organelles such as PM, ER, mitochondria, cytoskeleton and nucleus did not enrich (Fig. 3F,G). Ultrastructural analysis confirmed the high purity and virtual absence of contaminating organelles (Fig. 3H). As expected from SIM-CLEM, LE/LYS isolates from NPC1-KO cells had a clearly distinct morphology compared to those of WT cells comprising more pleiomorphic, larger organelles including multilamellar features (Fig. 3H).

### Lipidomic analysis on SPION-isolates

**Figure 4:**
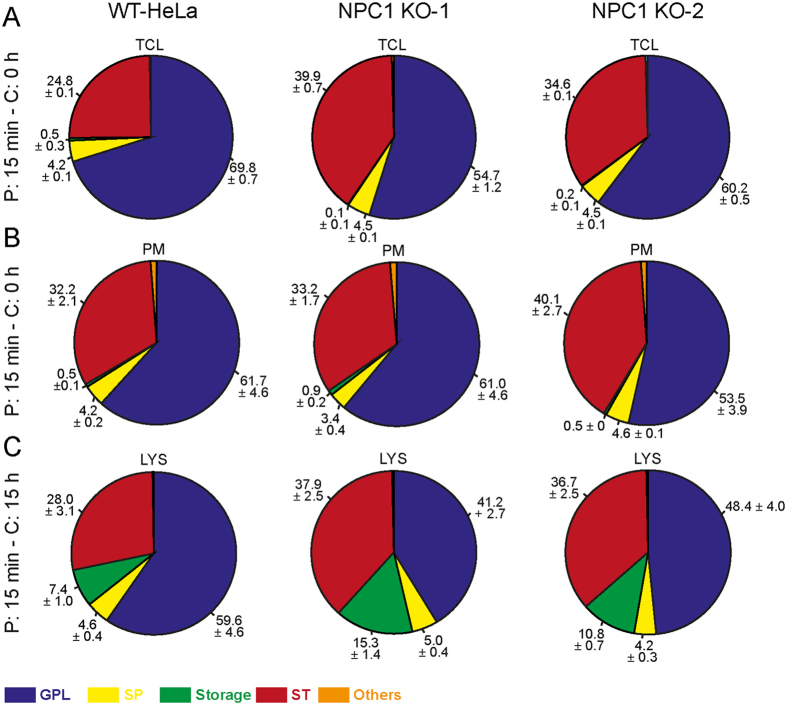
Storage lipids and sterols are markedly increased in LE/LYS isolated from NPC1-KO cells. Comparison of functional lipid categories of **(A)** total cell lysate (TCL), **(B)** PM and **(C)** LE/LYS from WT and NPC1-KO HeLa cells as determined by summing up absolute abundances of all identified lipid classes. Values are standardized to mole percentage within the sample. Glycerophospholipids (GPLs): phosphatidylcholine/-ethanolamine/-inositol/-serine/-glycerol (PC/PE/PI/PS/PG) and ether-linked PC/PE (PC O-/PE O-), phosphatidic acid (PA) and diacyl-glycerol (DAG). Sphingolipids (SP): ceramide (Cer), hexosylceramide (HexCer) and sphingomylein (SM). Storage: triacylglycerol (TAG) and cholesterol esters (SE). Cholesterol (ST), others: lyso- PC/PE/PI/PS/PA and ether linked PC/PE (LPC O-/LPE O-).

We next explored the lipidome of PMs and LE/LYS from WT and NPC1-KO cells using shotgun lipidomics. Lipids extracted from TCL, PMs and LE/LYS were subjected to LC-MS/MS along with lipid class-specific internal standards (see Methods) for absolute quantification of 17 lipid classes and 551 lipid species (Supp. Table S1). Data were analyzed using LipotypeXplorer and are presented as fold change of lipid species relative to WT. To get an overview of the changes between NPC1-KO cells and their WT counterparts, we divided functional lipid categories according to LIPID MAPS (LIPID Metabolites and Pathways Strategy) categories with slight modifications (Fig. 4). As expected, TCL of NPC1-KO cells had more cholesterol (ST) (>35 mol% in NPC1-KO compared to 25% in WT) in agreement with a cholesterol transport blockade due to NPC1 deficiency. Isolated PMs and LE/LYS showed distinct distributions of lipid categories. Whereas the PM lipidome minimally differed between WT and NPC1-KO cells, storage lipids such as triacylglycerol (TAG), cholesterol esters (SE) known to reside in lipid storage bodies were depleted from PMs (<1% compared to 2–3% in TCL) (Fig. 4B). On the other hand, Glycerophospholipids (GPLs) were reduced in LYS of NPC1-KOs (from 59.6 ± 4.6 mol% in WT to 41.2 ± 2.7 mol% in KO1, and 48.4 ± 4.0 mol% in KO2), while ST and storage lipids were clearly and more strongly enriched compared to WT LYS contributing, respectively, 37.9 ± 2.5 mol% and 15.3 ± 1.4 mol% in KO1 and 36.7 ± 2.5 mol% and 10.8 ± 0.7 mol% in KO2 (Fig. 4C).

**Figure 5:**
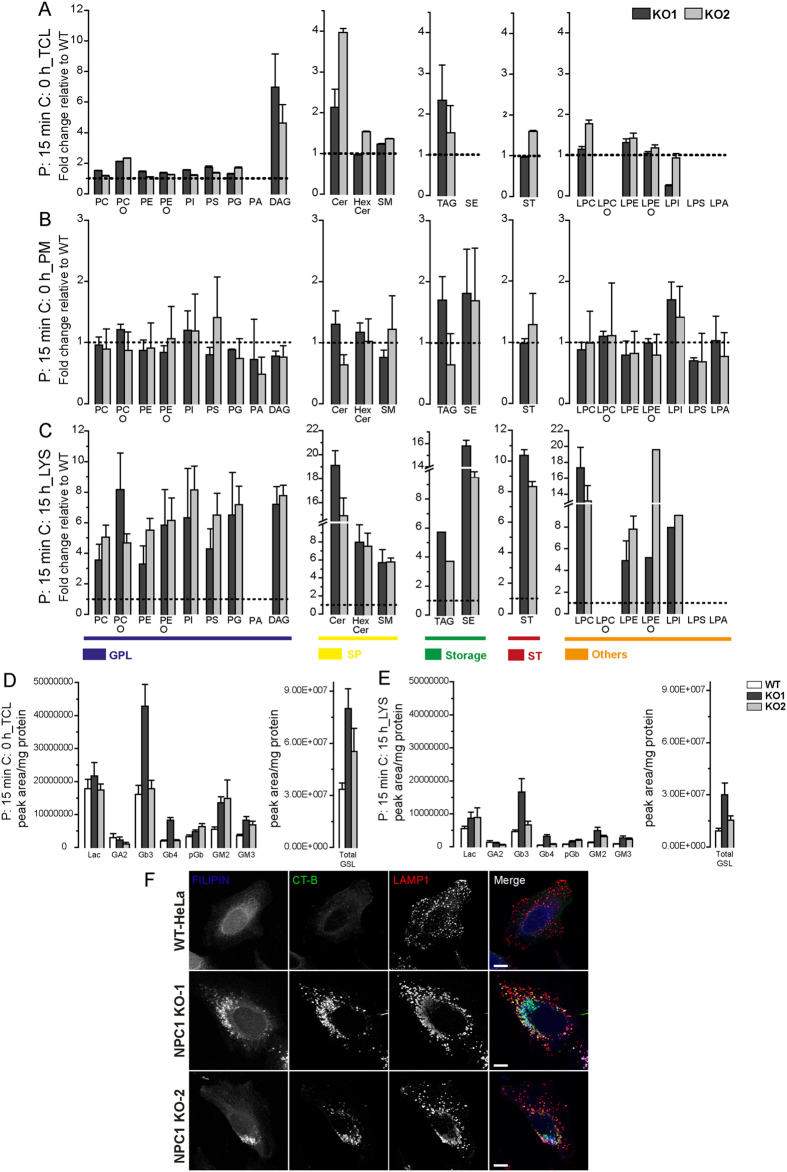
LE/LYS isolated from NPC1-KO cells contains elevated levels of several lipid classes. Enrichment plot showing lipid class compositions as relative fold change enrichments in TCL **(A)**, PM **(B)** and LE/LYS - chase 15 h **(C)** of NPC1-KO cells compared with WT cells as measured by MS. An increase in all major lipid classes was observed in LE/LYS of NPC1-KO cells, but not in PM or TCL. The content of individual lipid class was determined by summing up absolute abundance of all identified species and expressed as relative fold enrichment (mean ± SEM, n = 3). Glycospingolipid (GSL) levels are quantified using normal-phase high-performance liquid chromatography and expressed as peak area per milligram protein in TCL **(D)** and LE/LYS – chase 15 h **(E)** of WT and NPC1-KO cells. **(F)** Cholera toxin-B (CT-B) accumulates in filipin and LAMP1-positive organelles in NPC1-KO but not in WT-HeLa cells. Glycerophospholipids (GPLs): phosphatidylcholine/-ethanol a mine/-inositol/-serine/-glycerol (PC/PE/PI/PS/PG) and ether-linked PC/PE (PC O-/PE O-), phosphatidic acid (PA) and diacyl-glycerol (DAG). Sphingolipids (SP): ceramide (Cer), hexosylceramide (HexCer) and sphingomyelin (SM). Storage: triacylglycerol (TAG) and cholesterol esters (SE). Cholesterol (ST), others: lyso- PC/PE/PI/PS/PA and ether-linked PC/PE (LPC O-/LPE O-).

Further analysis of individual lipid species revealed additional differences. LYS of NPC1-KO cells had higher levels of most lipid species indicating an overall defect in lipid trafficking and metabolism: all GPLs including phosphatidylcholine (PC), phosphatidylethanolamine (PE), phosphatidylinositol (PI), phosphatidylserine (PS), phosphatidylglycerol (PG), and their ether-linked counterparts PC/PE (PC O-/PE O-) and diacylglycerol (DAG) showed over three-fold increases (Fig. 5C). Sphingolipids (SPs) such as ceramide (Cer), glucosyl- and galactosylceramides grouped as hexosylceramides (HexCer), and sphingomyelin (SM) were also significantly increased in LYS isolates of NPC1-KO cells (Fig. 5C). Since ceramide is known to displace cholesterol from membrane and membrane domains^61^,^62^ and since its concentration is inversely proportional to cholesterol concentration, the fact that both lipid species accumulated in LYS of NPC1-KO cells is surprising. Interestingly, lysophosphatidylcholines (LPC) were found to be strongly increased (>ten-fold) in LYS of NPC1-KOs along with lysophosphatidylethanolamine (-ether) (LPE-O). However, such differences were not observed in TCL (Fig. 5A) nor PMs (Fig. 5B) of NPC1-KOs underscoring that the change in the LYS lipidome is specific and caused by NPC1 deficiency. A closer look at structural details of lipid species in LYS and PMs did not reveal striking differences in GPLs (Supp. Fig. 7A–F and Supp. Table S2); however SPs in LYS of NPC1-KO cells were slightly longer, more saturated and more hydroxylated (Suppl. Fig. 7K–M). We next quantified glycosphingolipids (GSLs) using normal-phase high-performance liquid chromatography (NP-HPLC) (Fig. 5D,E). Ceramide (Cer) acts as precursor for GSL synthesis, and since its level was higher in TCL and LYS fractions of NPC1-KO cells, we predicted an increase in GSL levels as well. In agreement, total levels of GSL were significantly higher in TCL and LYS of NPC1-KO cells. A closer look at individual GSL-species revealed significant increases in TCL of NPC1-KO cells; however not in LYS fractions where the increase was only moderate. This was confirmed by staining with cholera toxin B (CT-B)-Alexa488 which selectively labels GM1 (Fig. 5F).

### Quantitative proteomics on SPION-isolates

**Figure 6:**
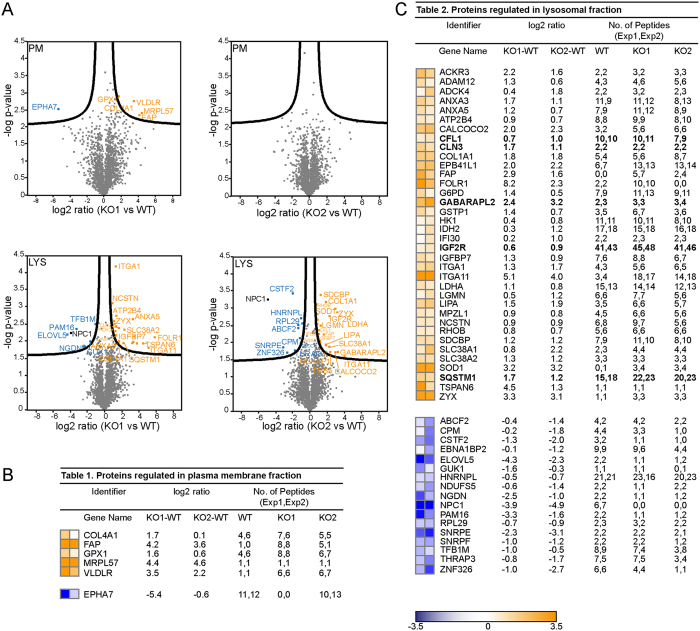
Proteome of LE/LYS differs whereas the PM proteome remains largely unaltered in NPC1-KO cells compared to WT cells. Volcano plot showing differentially regulated proteins as log2 ratios in PM and LE/LYS - chase 15 h (**A**) isolated from NPC1-KO cells compared to WT cells. Analysis of the log2 ratios of these proteins in NPC1-KO versus WT identified 6 proteins in PM **(B)** and 53 proteins in LE/LYS (**C**) that were significantly differentially expressed (for p-values see Supp. Table S3 and S4). Identified proteins are listed in Table 1 (**B**) and Table 2 (**C**) as heatmap along with their log ratios and peptide numbers by which they were identified.

**Figure 7:**
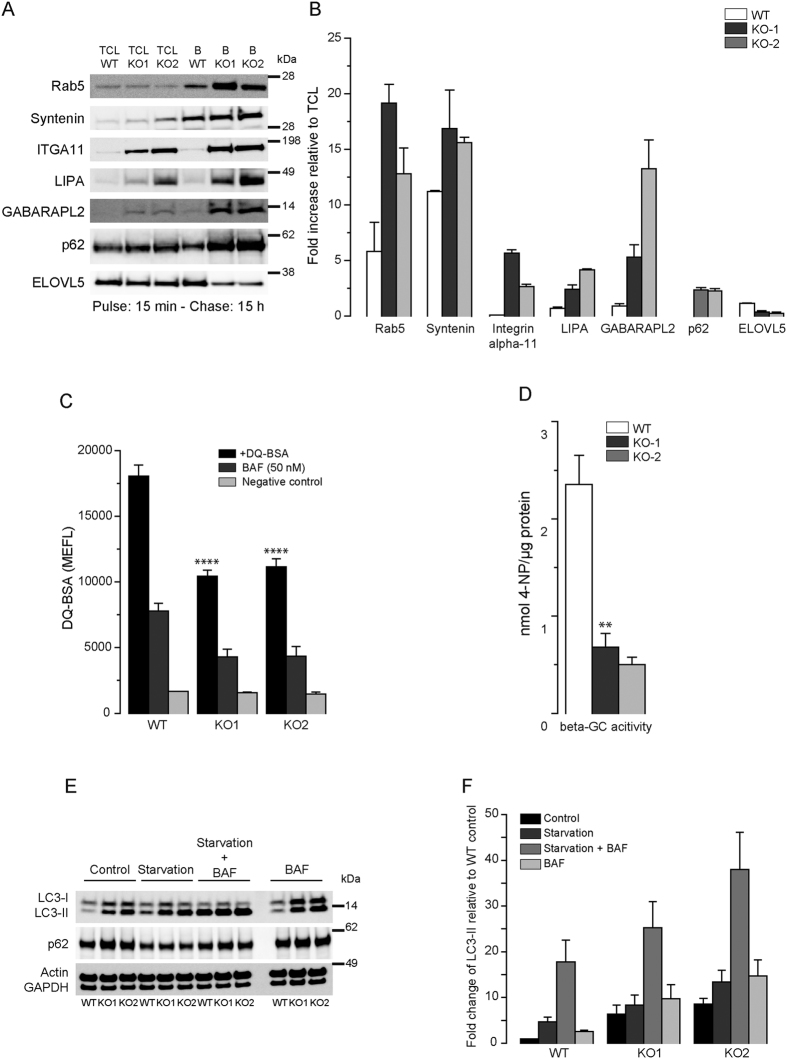
Impairments in lysosomal function and autophagosome maturation delays cargo clearance in NPC1-KO cells. (**A**) Western blot analysis of TCL and LE/LYS fractions for various proteins that were significantly up- or downregulated in NPC1-KO cells compared with WT HeLa as obtained by quantitative proteome analysis. (**B**) Quantification of the blots in (**A**) expressed as a fold increase relative to total cell lysate (TCL) (mean ± SEM, n = 3). NPC1-KO cells have strongly decreased lysosomal proteolytic (**C**) and β-glucocerebrosidase (beta-GC) activities (**D**). (**E**) Immunoblot analyses with anti-LC3 and anti-p62 antibodies shows upregulation of autophagy and defect in autophagic flux in NPC1-KO cells. (**F**) Quantification of (**E**) relative to actin (loading control), expressed as a fold change relative to WT (mean ± SEM, n = 3). Scale bar = 10 μm.

We next tested the suitability of our PMs and LE/LYS isolates for proteome analysis. Following digestion of the isolates, generated peptides were analyzed by LC-MS/MS and quantified by the MaxLFQ algorithm integrated in MaxQuant software (see Methods). We adopted label-free quantitative proteomics and compared PM and LYS composition of two NPC1-KO cells versus WT in duplicates. In each analysis, the LFQ values were log2 transformed and replicates grouped. Protein groups with less than two valid values in at least one group were removed and missing values were imputed with random values around the detection limit. This results in a fictive ratio for some proteins that were completely absent in one of the genotypes (either WT or NPC1-KO), which in turn prevents losing these potential interesting proteins during statistical analysis. Comparison of protein expression levels between WT and NPC1-KO cells was done with two-sample t-tests (FDR 0.05 and S_0_ 0.1) and protein ratios lying outside the limits were considered as significantly differentially regulated (Supp. Tables S3 and S4). In line with lipidomics, we observed marginal changes in the PM proteome between WT and NPC1-KO cells (Fig. 6A) as represented by the rather unimodal ratio distribution (Fig. 6A). This agrees with the view that major transport defects are at the level of intracellular trafficking though without majorly affecting the biomolecular composition of the cell surface. In contrast, and supporting this conclusion, the broad ratio distribution between LYS of WT and NPC1-KO indicates that a considerable number of proteins are differentially expressed in these organelles (Fig. 6A) and, like for lipid changes, are directly related to NPC1 deficiency. We further selected LYS proteins that were differentially expressed in NPC1-KO cells (Fig. 6B,C). This resulted in a heat map of 53 proteins, which revealed a group of 36 up-regulated and 17 down-regulated proteins in NPC1-KO LE/LYS. Among downregulated proteins we confirmed decreased levels of ELOVL5 by immunoblotting (Fig. 7A,B). ELOVL5 has been reported to locate to the ER and to be a rate-limiting enzyme in the production of monounsaturated and polyunsaturated very long chain fatty acids, which in turn act as precursors of several membrane lipids. Interestingly, ELOVL5 was found downregulated together with NPC1 (and other genes mediating cholesterol transport) in an Alzheimer mouse model treated with an anti-diabetes drug^63^. Also increased levels of annexin5 in LYS of NPC1-KO agrees with earlier reports demonstrating its presence on LE^64^ and increased abundance on LYS following starvation and its proposed role in autophagosome fusion with LYS^65^.

Interestingly, many lysosomal proteins such as LIPA, IFI30, IGF2R, CLN3, LGMN, autophagy-related proteins (e.g. GABARAPL2, CALCOCO2, SQSTM1) as well as endosomal proteins (FOLR1, ACKR3, RHOB) were found to be upregulated in NPC1-KO LE/LYS. However certain proteins were found to be significantly more or less present in either PM or LE/LYS in only one of the KO cell lines and hence were excluded from the set of regulated proteins. This could originate from the biological variability inherent to the different generated clones. Hence, for future applications, it is recommended to either use more independent clones or, alternatively, to compare KO of the respective protein in distinct cell lines in order to identify with higher stringency common dysregulated proteins. Although limited by available antibodies, we could show by quantitative immunoblotting that several of these proteins are indeed more enriched in NPC1-KO LYS (Fig. 7A,B). Since we find notable changes in expression of several lysosomal enzymes such as RNASET2, IFI30, ARSA, LIPA in our proteomics data, we checked proteolytic and enzymatic activities of LYS in WT and NPC1-KO cells using DQ-BSA and β-glucocerebrosidase (Fig. 7C,D). Despite higher expression, LYS of NPC1-KO cells have lower levels of enzymatic activity along with impairment in the proteolytic function. Several proteins that are upregulated belong to the CLEAR pathway of autophagolysosomal degradation^66^. This includes for instance GABARAPL2, a member of the GABARAP family, that functions in autophagosome maturation, particularly in its elongation and closure^67^,^68^, RAB5A (endocytosis), CLN3, IGF2R (lysosomal biogenesis), SQSTM1 (autophagy), CFL1 (regulation of actin cytoskeleton)^69^. Also our TEM data demonstrated an abundance of autophagic organelles (Fig. 3A,B) indicating that autophagy in general is upregulated in NPC1-KO cells. LC3, and in particular the conversion from LC3-I to the lipidated LC3-II is widely used to monitor autophagic flux^70^. LC3-II levels are indeed constitutively increased in NPC1-KO and further increased following starvation and in combination with Bafilomycin which blocks lysosomal fusion (Fig. 7E,F).

## Discussion

In this study, we demonstrated that thermal decomposition combined with different surface functionalizations can be successfully used to synthesize SPIONs for subsequent applications in isolating distinct subcellular compartments. More particularly, we generated aminolipid- and DMSA-SPIONs that selectively target PMs and LE/LYS. We argue that the preferred cell surface localization of aminolipid-SPIONs might be due to lipid-lipid interaction and/or charge-dependent electrostatic interactions which might halt or at least greatly decrease cellular uptake, as observed with magnetoliposomes^71^. Uptake of anionic DMSA-SPIONs is likely mediated through nonspecific adsorption of serum proteins onto the SPIONs’ coating, as reported for anionic gold nanoparticles^34^.

The selectivity of targeting distinct membrane compartments is strongly underscored biochemically and through TEM analysis on both fixed cells and isolated fractions. The aminolipid-SPIONs provide a superior alternative to the cell surface biotinylation approach^72^ and the anionic DMSA-SPIONs, in contrast to those reported earlier^73^,^74^, are more stable, show less cytotoxicity and hence provide less risk for use in cellular uptake and isolation experiments. Since the biophysical characteristics of PMs and endosomal compartments are more dynamic and variable, such compartments are very difficult to purify reproducibly from different sources using traditional methods based on gradient centrifugation. Immunoaffinity approaches might provide an alternative but are limited to available antibodies, the restricted localization and relative abundance of the respective antigens on the organelle of interest. Our methodology obviates the need for any kind of classic affinity labeling procedures or detergents and can be performed in a relatively short experimental time window. The high recovery of intact PM and LE/LYS fractions along with their high purity provide a gateway to profile the overall biomolecular composition of these organelles using subsequent ‘omic’ approaches. As such, we have validated their analysis in subcellular omics by comparing the lipid and protein profiles of PMs and LE/LYS isolated from WT HeLa and HeLa cells deficient for NPC1 expression, as an *in vitro* model for NPC.

Although NPC is perceived as a cholesterol storage disorder, recent studies have dissected out the chronology of events starting with sphingosine accumulation leading to alterations in calcium homeostasis and downstream accumulation of cholesterol and sphingolipids^51^. Calcium also plays a vital role in endocytic events and its insufficient release from acidic compartments such as LE/LYS has been shown to affect the fusion of incoming maturing LE and autophagic vacuoles with LYS^75^. This delay in lysosomal fusion is also observed in our SIM-CLEM analysis. While in normal WT cells, DMSA-SPIONs reach their final destination in lysosomes already after 4 h, in NPC1-KO cells, they labeled a pool of LE/LYS very distinct from the pleiomorphic and filipin-positive organelles, the latter likely representing older autophagolysosomes. More extended incubation periods were needed to chase these particles ultimately to this pool underscoring that fusion is not completely blocked but significantly delayed. This could be mechanistically explained by the observation that lysosomal accumulation of SMs, as occurs in NPC1-deficient cells (this study)^51^, inhibits the activity of the principle Ca^2+^ -channel, TRPML1, thereby disturbing lysosomal Ca^2+^ homeostasis and trafficking/fusion events^76^. In a proteomics study on whole cell lysates from WT and NPC1-KO MEFs, Sarkar and colleagues found several proteins relating to autophagy as being regulated. They further went on to show that NPC1-KO cells failed to recruit proteins of the SNARE machinery to LE thereby blocking their fusion and maturation into autophagosomes^77^. However, very few studies have focused on analyzing the global changes, with respect to both proteins and lipids, occurring in the defective lysosomal compartments caused by NPC1 deficiency. An unbiased profiling of such components enables, therefore, the identification of perturbed pathways and mechanisms, as shown here for the autophagic pathway, which contributes to the manifestation of complex diseases such as NPC.

To date, only a few such profiling studies for NPC have been reported^78^,^79^ and most of these studies represent only the “tip of the iceberg” as the enormous biomolecular complexity, and its dynamic range of concentrations in a cell/tissue extract can still not be fully resolved. Organellar omics studies as outlined here increase the chance of detecting low abundant molecules enabling the study of disturbed organellar dynamics. Our unique lipidomic profiling of isolated LE/LYS and TCL of WT and NPC1-KO cells substantiates this statement. Quantitative lipidomic analysis of LE/LYS isolated from WT and NPC1-KO cells revealed significant changes in the lipidome, however such changes were not detected in TCL of the same cells. Although the accumulation of cholesterol has been seen as a primary hallmark of this lipid storage disorder, we show here an accumulation of other lipids including GPLs, GSLs and other storage lipids such as Cer, HexCer and SM in LE/LYS of NPC1-KO cells and not in WT. These lipidome alterations are essentially not observed in the lipid profiling of isolated PMs, but recapitulated in LE/LYS isolates suggesting a more selective alteration of membrane composition in the degradative route. Thus subcellular lipidomics adds spatial information to the analysis, which is not reached when total cells or tissues are analyzed. Furthermore, the absence of NPC1 resulted in significant upregulation of 36 proteins, including many proteins involved in autophagy and the catabolic functions of lysosomes. Not surprisingly, we found alterations in both pathways by independent assays. These findings overall underscore the severe defects in lysosomal turnover that is likely the result of a cascade of events emanating from a defect in calcium signaling from acidic stores blocking fusion and transport of lipid substrates in LE/LYS. Not surprisingly, we found a constitutive upregulation of autophagy as there is a failure in their fusion with LE/LYS.

In this study, we have demonstrated that functionalized SPIONs are a promising and efficient resource tool for organelle isolation that is moreover compatible with subsequent omics analysis. Given the lower complexity of an organelle compared to a whole cell or tissue, omics analysis provides a higher resolution. We furthermore validated that a combined analysis of the lipidome and proteome additionally allows for a better evaluation of the global changes occurring in specific organelles, in this case, PMs and LE/LYS as a consequence of disease-related protein deficiencies. The focus on PMs and LE/LYS using the same isolation and analysis approach is particularly interesting given the fact that hitherto, a major part of the know drugs target surface receptors and proteins, and lysosomal dysfunctions are becoming more and more a central theme in human diseases, including neurodegenerative diseases and cancer^80^,^81^,^82^. The increased resolution provided by subcellular omics on SPION-isolated PM and LE/LYS may facilitate identification of protein and lipid alterations at the cell surface (e.g. related to cellular reprogramming during cancer progression, neuronal differentiation, and immune responses) or aberrantly accumulating cargo in lysosomal storage disorders as well as neurodegenerative diseases. Such strategies are important in experimental medicine as they would increase the probability to identify signaling pathways that become dysregulated in disease, protein targets as well as potential biomarkers.

## Methods

### Reagents

Iron (III) acetylacetonate (≥99.9%), 1,2-hexadecanediol (technical grade, 90%), oleic acid (90%), benzyl ether (90%), potassium bromide (≥99%), dimercaptosuccinic acid (DMSA) (~98%), dimethyl sulfoxide (DMSO) (>99.9%), ammonium bicarbonate (≥99.5%), and n-hexane (>99.0%) were purchased from Sigma-Aldrich (Steinheim, Germany), while oleylamine (>70%) and extra-dry toluene were obtained from Acros (Geel, Belgium). Ethanol was purchased from Honeywell (Sleeze, Germany) and chloroform from Merck (Darmstadt, Germany). The phospholipids 1,2-distearoyl-sn-glycero-3-phosphoethanolamine-N-[carboxy(polyethylene glycol)-2000] (Carboxylipid), 1,2-distearoyl-sn-glycero-3-phosphoethanolamine-N-[methoxy(polyethylene glycol)-2000] (Methoxylipid) and 1,2-distearoyl-sn-glycero-3-phosphoethanolamine-N-[amino(polyethyleneglycol)-2000] (Aminolipid) were from Avanti Polar Lipids (Alabaster, AL, USA). Dulbecco’s Modified Eagle Medium: Nutrient Mixture F-12 (DMEM/F12), Fetal Bovine Serum (FBS) and Phosphate Buffered Saline (PBS), Coomassie G-250 (SimplyBlue™ SafeStain), Earle’s Balanced Salt Solution (EBSS) were obtained from Invitrogen (Belgium). MACS LS columns were purchased from Miltenyi Biotec B.V. (Leiden, The Netherlands), acetonitrile from Baker (HPLC grade, Deventer, The Netherlands), Filipin III - *Streptomyces filipinensis* from Sigma and trypsin from Promega (WI, USA).

### Antibodies

The following monoclonal antibodies (mabs) were obtained from commercial vendors: anti-actin, anti-EEA1 (Sigma), anti-Na^+^K^+^-ATPase (Novus-Biologicals), anti-GAPDH (Millipore), anti-human Lamp-1 (BD Transductions), anti-Integrin alpha 11 (R&D system), anti-LC3B (Cell Signaling), anti-transferrin receptor (TfR, clone H68.4; Invitrogen), anti-Lamin and anti-Cathepsin D (Santa Cruz biotechnology). A mab against Rab5 was provided by R. Jahn (Max Planck Institute, Gottingen, Germany). Polyclonal antibodies (pabs) against p58 and Ribophorin (RBI) were provided by R. Schekman (Berkeley), anti-RAB7 by P. Chavrier (CNRS, Paris), anti-PEX14p from M. Fransen (KU Leuven, Belgium) while pabs against NPC1 (abcam), BiP (Cell Signaling), PARP (Cell Signaling), Caspase3 (Cell Signaling), ELOVL5 (Sigma), p62 (Sigma), GABARAPL2 (proteintech) and Lipase A (Novus-Biologicals) were obtained commercially.

### Cell lines

All *in vitro* cellular experiments were performed on human cervical cancer cells (HeLa) which were cultured in DMEM/F-12 supplemented with 10% FBS according to established procedures.

### Generation of NPC1 Knockout (KO) HeLa cells

Genomic target for NPC1 ((Exon-2: 5′-AGGTACAATTGCGAATATTC-3′) and (Exon-4: 5′-AAAGAGTTACAATACTACGT-3′)) was selected using CRISPR design tool (http://www.genome-engineering.org/crispr/). The gRNAs were annealed and ligated into pX330 plasmid (Addgene) as described^83^. Selection of KO clones was done by serial dilution and Western blot analysis.

### Synthesis of magnetic nanoparticles

Thermal decomposition was performed as described^84^ with some modifications (Supp. Fig. S1A). Briefly, 2 mmol of iron (III) acetylacetonate, 10 mmol of 1,2-hexadecanediol, 6 mmol of oleic acid and 6 mmol of oleylamine were mixed with 20 ml benzyl ether under continuous nitrogen flow and heated to 200 °C for 2 h in a reflux setup. Following this, the mixture was further heated to reflux (~300 °C). After 1 h at reflux, the mixture was allowed to cool down to room temperature. Under ambient conditions the SPIONs were washed three times with ethanol while retaining the SPIONs using a rare earth magnet. Finally, the SPIONs were suspended in n-hexane, centrifuged for 10 min to remove major aggregates and residues, and kept at 4 °C until further use.

### PEGylated phospholipid functionalization

Aminolipid-coated SPIONs were formed by adopting a procedure originally described for water-dispersible quantum dots^52^. Hereto, 5 mg of SPIONs were dispersed in 1 ml of chloroform and 10 mg of aminolipid was added. The mixture was left under continuous agitation for 4 h at room temperature. Following this, the chloroform was evaporated under a flow of nitrogen and 1 ml of distilled water was added to the dried SPIONs. After 5 min of vigorous shaking, a uniform transparent SPIONs-containing aqueous solution was formed. The dispersion was further centrifuged for 10 min to remove any major aggregates. Further purification was performed by trapping and washing the SPIONs on a LS column (Miltenyi). The bound fraction was finally re-suspended in 1 ml of phosphate buffered saline (PBS). A similar protocol was used for the functionalization of SPIONs with the carboxylipid- or methoxylipid-coated SPIONs.

### DMSA functionalization

DMSA-functionalized SPIONs were manufactured as described previously^85^. In short, 10 mg of SPIONs were suspended in 1 ml of extra-dry toluene and mixed with 200 mg of DMSA dissolved in 1 ml of DMSO. The mixture was allowed to react at room temperature for 72 h under rigorous agitation. Subsequently, the mixture was centrifuged to remove any un-reacted DMSA and DMSO and the pellet was re-suspended in 1 ml of distilled water and separated on a LS column. In a final step the bound fraction was dispersed in 1 ml of distilled water and the pH adjusted to 7.

### Magnetic Nanoparticle characterization

#### Transmission electron microscopy (TEM)

TEM samples were prepared by drop casting a diluted suspension of SPIONs on an amorphous carbon-coated copper grid and allowing the solvent to evaporate at room temperature. TEM images were captured using a 300 kV CM30 instrument (Philips, Eindhoven, The Netherlands) equipped with a field emission gun electron source. A statistical analysis was carried out by measuring the diameter of at least 200 nanoparticles.

#### Thermal gravimetric analysis (TGA)

TGA was performed on a Q5000 IR (TA instruments, New Castle, DE, USA) under nitrogen atmosphere to determine the mass concentration of the SPIONs. 100 μl of SPIONs were placed on a pan and slowly heated to 80 °C to remove all solvents. Next, the temperature was increased to 850 °C with a heating rate of 20 °C/min. Following the evaporation of the coating the amount of iron was calculated using the software provided by the manufacturer (Universal Analysis 2000, v 4.5A).

#### Dynamic Light Scattering (DLS) and zeta-potential

The hydrodynamic size and zeta-potentials of the SPIONs at various pH with and without functionalization were measured using a Zetasizer NanoZS instrument (Malvern, Leusden, The Netherlands) equipped with a red laser (633 nm) in backscatter mode (173°). The hydrodynamic diameter was measured from a dilute suspension of the sample in water in a plastic cuvette at pH 7. The zeta- potential was also measured in a special zeta-potential cuvette from a dilute suspension of the sample and taken at different pH. The hydrodynamic diameter and the surface charge of the SPIONs were examined using the Zetasizer software v.6.01 provided by the manufacturer.

#### Fourier-transform infrared spectroscopy (FTIR)

300 mg of KBr and approximately 1 mg of SPIONs before or after functionalization were mixed. This mixture was dried in an oven (under vacuum) for 48 h at 50 °C and subsequently pressed to form a thin pellet, which was mounted in a IFS66/v FTIR device (Bruker Optik GmbH, Ettlingen, Germany). A pure 300 mg KBr pellet was used as background and subtracted from the FTIR spectra of the SPMNP samples. Data were collected using 2048 scans between 4000 and 500 cm^−1^ at a resolution of 1 cm^−1^. The organic composition of the SPIONs was investigated with OPUS software (v5.0).

#### Vibrating Sample Magnetometer (VSM)

Hysteresis magnetization measurements were performed to study the magnetic properties of the SPIONs using a VSM (Oxford Instruments, UK). The hysteresis curves were measured at 300 K and fitted with a Langevin function using Origin 8.1.

### Cytotoxicity assay

Following the incubation at indicated time points at 37 °C with surface-functionalized SPIONs (DMSA, aminolipid, carboxylipid or methoxylipid-coated) the cells were trypsinized, mixed thoroughly with trypan blue (1:1) and the number of live and dead cells were counted using a Countess II automated cell counter (Thermo Fisher Scientific).

### ER stress assay

HeLa cells were incubated for the indicated time at 37 °C with aminolipid-/DMSA-coated SPIONs or with 2 μM thapsigargin. Following incubation, cells were washed, harvested in PBS, centrifuged (180 g, 10 min) and lysed. The protein concentrations were measured and equal quantities of denatured samples were separated on a pre-cast 4–12% gradient Bis-Tris gel (Invitrogen) followed by immunoblot analysis for BiP. Complementary, RT-PCR for XBP1 splicing was performed as described previously^86^. RNA was isolated from the cells using the High Pure RNA Isolation Kit (Roche Applied Science, 11828665001), followed by cDNA synthesis with the High-Capacity cDNA Reverse Transcription Kit (Life Technologies, 4368814).

### Cell Death Measurement

Following incubation with aminolipid- or DSMA-coated SPIONs at 37 °C for the indicated time points, cells were trypsinized, mixed thoroughly with propidium iodide (1 μg/ml) and the percentage of apoptotic cells was determined using a FACSAria III instrument. Alternately HeLa cells incubated with aminolipid-/DMSA-coated SPIONs for the indicated time at 37 °C were harvested in PBS, centrifuged (180 g, 10 min) and lysed. Equal amounts of protein were analysed by SDS-PAGE on pre-cast 4–12% gradient Bis-Tris gels (Invitrogen) followed by immunoblot analysis for PARP. Cells treated with Bortezomib (100 nM) for 24 h at 37 °C were taken as a control.

### DQ-BSA Proteolytic assay

DQ-green-BSA was used as an artificial substrate to evaluate lysosomal proteolytic degradation. Briefly, WT and NPC1-KO HeLa cells were incubated with DQ-green-BSA (5 μg/ml) at 37 °C for 1 h or combined with DMSA-coated SPIONs for 4 and 15 h at 37 °C. Following the incubation, cells were trypsinized, washed and resuspended in PBS (containing 1% BSA, 2% FBS) and assessed on an Attune flow cytometer wherein a minimum of 10 000 positive events were recorded. Cells treated with bafilomycin A1 (100 nM) were taken as a control.

### Transferrin uptake

HeLa cells plated on coverslips were incubated with DMSA-coated SPIONs (pulse 15 min, chase 4 h at 37 °C). Next, cells were incubated for 30 min at 4 °C in the presence of Alexa568-conjugated transferrin(Tf-568, 50 μg/ml in serum-free medium). Following a brief wash with ice cold serum-free medium, cells were incubated at 37 °C for 15 min to allow internalization of receptor bound Tf-568. Finally, cells were washed with ice-cold PBS followed by an acidic rinse (0.2 M glycine, pH 3), fixed on ice for 20 min (4% PFA) and finally processed for immunofluorescence microscopy.

### Cell Surface Biotinylation

HeLa cells were incubated with DMSA-coated SPIONs (pulse 15 min and chase 4 h at 37 °C). Next, cell surface biotinylation was performed as described^87^. Untreated cells were used as a control and percentage of internalized transferrin receptor was quantified between the two conditions.

### Analysis of autophagosome formation

Autophagosome synthesis was analyzed by measuring LC3-II levels relative to actin (loading control). WT and NPC1-KO HeLa cells under basal or starved condition (4 h with EBSS) were harvested in PBS, centrifuged (180 g, 10 min) and lysed. The protein concentrations were measured and equal quantities of denatured samples were separated on a pre-cast 4–12% gradient Bis-Tris gel (Invitrogen) followed by immunoblot analysis for LC3 and p62. As a control, cells treated with 100 nM bafilomycin A1 for the last 1 h prior to harvesting the cells for immunoblot analysis were taken.

### Measurement of relative lysosomal volume

WT and NPC1-KO HeLa cells were grown in T175 culture flasks, trypsinized, centrifuged (180 g, 10 min), washed with 1xPBS, centrifuged again and 0.5*10E6 cells in duplo were stained with 1 ml of 200 nM LysoTracker-green DND-26 (Invitrogen) in PBS (10 min, in dark). Following incubation, cells were centrifuged (800 g, 5 min), resuspended in 0.5 ml of FACS buffer (0.1% BSA, 0.02 M NaN_3_ in 1 × PBS) and kept on ice for a maximum of 1 h prior to flow cytometric analysis (BD Biosciences FACSCanto II). Mean equivalent of fluorescence (MEFL) was calculated using 8-peak Rainbow calibration beads (BD) using the fluorescein-equivalent values provided by the manufacturer.

### Pulse chase methodology and magnetic isolation

Internalization of SPIONs was studied in HeLa cells grown to near 90% confluency on 10-cm culture dishes. Briefly, cells were incubated (*Pulse*) for 15 min at 37 °C with aminolipid-, carboxylipid-, or methoxylipid-coated SPIONs at a concentration of 2 mg/ml or with DMSA-coated SPIONs at concentration of 200 μg/ml in culture medium. After washing in PBS, cells were re-incubated (*Chase*) at 37 °C in fresh medium devoid of nanoparticles for 0 to 4 h or 15 h. At the end of the chase period (internalization), the cells were washed three times with acidic buffer (0.15 M glycine, pH 3), cold PBS and processed for cell fractionation.

### Magnetic isolation

Cells were washed and harvested in PBS, centrifuged (180 g, 10 min) and homogenized in homogenizing buffer (HB; 250 mM sucrose, 5 mM Tris and 1 mM EGTA pH 7.4 supplemented with protease inhibitors) using a ball-bearing cell cracker (12 passages, clearance 10 μm, Isobiotec, Germany) to obtain a total cell lysate (TCL). After low-speed centrifugation (800 g, 10 min), the post-nuclear supernatant was loaded on a HB-equilibrated LS column placed inside a strong magnetic field (SuperMACSII separation system, Miltenyi). The LS column is packed with a hydrophilic coated matrix that strongly enhances the magnetic field and thereby ensures a more efficient retainment of the SPIONs (and adhering subcellular compartments) on the column. Within the magnetic field, the nonmagnetic fraction is first removed, followed by extensive washes with ice-cold HB to maximally remove any unbound material. Next, the magnet was removed and the bound fraction eluted using HB buffer. Following a high speed ultracentrifugation (126,000 g, 1 h) the resulting pellet was re-suspended in 200 μl HB and subjected to further analysis. Protein concentration was measured using the Bradford assay (Bio-Rad) and equal quantities (of TCL and B) denatured in sample buffer (Invitrogen) prior to separation on a pre-cast 4–12% gradient Bis-Tris gel (Invitrogen) followed by silver staining. Western blot analysis was performed and prior to primary antibody incubation the blots were cut along the molecular weight ranges of the respective antigens. Immunoreactive bands were detected using ECL (PerkinElmer) and digitally imaged on a Fuji MiniLAS3000. Quantification was done using Aida software on images with unsaturated exposures (Raytest, Germany). Starting from four culture dishes, PMs and LE/LYS isolates yielded on average 70–120 μg and 80–120 μg of protein.

### Transmission electron microscopy (TEM) on cells and isolated sub-compartments

HeLa cells grown on culture dishes to near confluency were incubated with functionalized SPIONs for 15 min at 37 °C. Subsequently the cells were washed once with PBS and the internalized SPIONs were chased for 0 to 4 h at 37 °C in a fresh medium. After magnetic labeling, cells were washed with acidic buffer (0.15 M glycine, pH 3), PBS and fixed using 2.5% glutaraldehyde in 0.1 M sodium-cacodylate buffer at 4 °C (overnight). Fixed cells were scraped in the presence of 0.1 M sodium-cacodylate buffer and centrifuged (200 g, 10 min at 4 °C). The pellets were resuspended in 1.5% agarose and incubated for 30 min at 4 °C. The embedded pellets were post-fixed in 1% osmium tetroxide (2 h), rinsed with dH_2_O, and dehydrated in a graded ethanol series (50–100%). Samples were *en bloc* stained with uranyl acetate in the 70% ethanol step for 30 min at 4 °C. Finally, after propylene oxide treatment (2 changes 15 min each), pellets were infiltrated with and embedded in 100% epoxy resin for two days (60 °C). Ultrathin sections of 50 nm thickness were examined and post-stained with 3% uranyl acetate in water (10 min), Reynold’s lead citrate (2 min). Micrographs were taken in a JEOL JEM2100 (JEOL, Japan) at 80 KV. Ultrastructural analysis of isolated PMs and lysosomes was done as described^88^.

### Correlative structured illumination microscopy and electron microscopy (SIM-CLEM)

WT and NPC1-KO cells grown on glass bottom dishes (MatTek) were incubated with DMSA-coated SPIONs for 15 min at 37 °C. Subsequently the cells were washed once with PBS and the internalized SPIONs were chased for 4 h and 15 h respectively at 37 °C in fresh medium. After magnetic labeling, cells were washed with acidic buffer (0.15 M glycine, pH 3), PBS and fixed using 1.25% glutaraldehyde in 0.1 M sodium-cacodylate buffer for 1 h at room temperature. Further, cells were washed 3× (10 min each) with PBS and filipin (200 μg/ml) staining was performed under light-protected conditions (2 h, RT).

### Structured illumination microscopy (SIM)

Images (transmitted light and fluorescence z-stack) of filipin staining were acquired on an inverted Zeiss Elyra S1 SIM (equipped with a stage for mounting dishes) using oil-immersion Plan-APOCHROMAT 63× objective lenses with 1.4 NA using ZEN 2011 software. During imaging, cells were maintained in PBS and for correlation purposes, marks were made on the bottom of glass dishes. Subsequently the PBS was replaced with 2.5% glutaraldehyde in 0.1 M sodium cacodylate buffer at 4 °C (overnight).

### TEM

Briefly, cells were washed 3x with 0.1 M sodium cacodylate buffer, post-fixed in 1% osmium tetroxide (2 h), rinsed with dH_2_O, and dehydrated in a graded ethanol series (50–100%). Samples were *en bloc* stained with uranyl acetate in the 70% ethanol step for 30 min at 4 °C. Following dehydration, cells were infiltrated with resin (Agar 100)/ethanol mixtures. The next day, cells were infiltrated and embedded with 100% epoxy resin in inverted BEEM-capsules for two days (60 °C). Ultrathin sections of 50 nm thickness were cut following the separation of polymerized cells from the glass bottom dishes (via freeze-thaw approach) and post-stained with 3% uranyl acetate in water (10 min) and Reynold’s lead citrate (2 min). Micrographs were taken in a JEOL JEM1400 (JEOL, Japan) equipped with an Olympus SIS Quemesa 11 Mpxl camera at 80 KV.

### Correlation

Light and electron microscopic images were overlaid using morphological signatures and correlated by overlaying one Z-plane of the fluorescence image with one Z-plane of EM image. The light and electron microscopic images were scaled and aligned using GIMP software.

### Confocal microscopy

Cells were fixed (4% paraformaldehyde, 30 min at RT), permeabilized (0.1% Triton X-100, 5 min at RT), blocked (2% goat serum, 60 min at room temperature) and processed for indirect immunofluorescence. Following overnight primary antibody incubation (4 °C), cells were incubated (60 min, RT) with Alexa Fluor 488-, Alexa Fluor 568-, or Alexa Fluor 647-conjugated secondary antibodies (Invitrogen) and mounted using Mowiol. Images were captured on a confocal laser microscope system (Nikon A1R) connected to an inverse microscope (Ti-2000; Nikon) or on a Leica TCS SP5 II (Leica Microsystems) connected to an upright microscope, using an oil-immersion plan Apo 60 × A/1.40 NA objective lens. Data collected using Nikon imaging software or LAS (Leica Microsystems) were further processed with ImageJ and PhotoshopCS6 (Adobe, CA). Co-localization analysis using Pearson’s coefficient was calculated on images obtained under identical microscopy settings using ImageJ with JACoP plugin^89^ and the data were represented as Mean ± SEM of at least 30 cells from two independent experiments.

### Lipidomics

#### MS/MS analysis on lipids extracted from TCL, isolated plasma membranes (PM) and late endosomes (LE)/lysosome (LYS)

Lipid analysis was performed by Lipotype (Dresden, Germany). Lipids were extracted from TCL, and PM and LE/LYS isolates using a two-step lipid extraction^90^ and subjected to mass spectrometric analysis. Prior to lipid extraction all the samples were spiked with lipid class-specific internal standards.

#### Spectra acquisition and data processing

Equal volumes of extracted lipids were injected from each sample and the mass spectra were acquired on a hybrid quadrupole/Orbitrap Q Exactive mass spectrometer (Thermo Fisher Scientific) equipped with an automated nano-flow electrospray ion source (Triversa Nanomate, Advion) in both positive and negative ion mode. Lipid identification was performed on unprocessed mass spectra using LipotypeXplorer^91^. In case of MS-only spectra, lipid identification was based on the molecular masses of the intact molecules while for MS/MS spectra, lipid identification was based on both the intact masses and the masses of the fragments. Lipid identifications were filtered according to spectral noise, background signals, mass accuracy and occupation threshold prior to normalization and further analysis. Lists of identified lipids and their intensities were stored in databases and quantification was based on lipid class-specific internal standards. For data handling and normalization, modules and tools implemented into the Lipotype LIMS was used. Values are expressed as fold change relative to the control samples (WT or TCL).

#### Glycosphingolipid measurement

Glycosphingolipid (GSLs) were isolated and analyzed essentially as described^92^. Briefly lipids were extracted from TCL and LYS isolated from WT, NPC1-KO HeLa cells using chloroform/methanol and GSLs were separated using solid phase C18 columns (Telos, Kinesis, UK). Eluted GSLs were dried under nitrogen and digested overnight with ceramide glycanase (prepared in-house from medicinal leeches (*Hirudo medicinalis*)). The released glycans were further fluorescently labelled with anthranilic acid (2AA), purified using DPA-6S amide columns (Supelco, PA, USA) and were separated, quantified by normal-phase high-performance liquid chromatography (NP-HPLC).

### Proteomics

#### MS/MS analysis on trypsin digests of TCL, isolated PMs and LE/LYS

##### Gel-based proteomics

10 μg protein of TCL, isolated PM and LE/LYS samples were separated by SDS-PAGE on a 4–12% gradient Bis-Tris pre-cast gel, followed by staining with SimplyBlue™ SafeStain (Invitrogen). Each lane was cut into 16 equally sized slices, which were subsequently washed with distilled water, 50% and 100% acetonitrile (ACN) for 15 min each. The gel slices were vacuum dried and re-suspended in 200 μl of 50 mM ammonium bicarbonate buffer containing trypsin (see further). The gel slices were let to re-swell and, if needed, additional buffer was added to fully submerge them. The final trypsin concentration was typically 5 ng/μl and digestion took place overnight at 37 °C. The generated peptide mixtures were centrifuged at 15,700 g for 5 min to remove possible debris. Peptides were collected, vacuum dried and re-dissolved in 15 μl of 2% acetonitrile and 0.1% TFA.

Of each sample, 5 μl was introduced into an LC−MS/MS system through an Ultimate 3000 RSLC nano LC (Thermo Fisher Scientific, Bremen, Germany) in-line connected to a Q Exactive mass spectrometer (Thermo Fisher Scientific). The sample mixture was first loaded on a trapping column (made in-house, 100 μm internal diameter (I.D.) ×20 mm, 5 μm beads C18 Reprosil-HD, Dr. Maisch, Ammerbuch-Entringen, Germany). After flushing from the trapping column, the sample was loaded on an analytical column (made in-house, 75 μm I.D. ×150 mm, 5 μm beads C18 Reprosil-HD, Dr. Maisch) packed in the needle (PicoFrit SELF/P PicoTip emitter, PF360-75-15-N-5, New Objective, Woburn, MA, USA). Peptides were loaded with loading solvent (0.1% TFA in water) and separated with a linear gradient from 98% solvent A’ (0.1% formic acid in water) to 40% solvent B’ (0.1% formic acid in water/acetonitrile, 20/80 (v/v)) in 130 min, at a flow rate of 300 nL/min. This was followed by a 15 min wash reaching 99% solvent B’ (see below for LC-MS/MS, data analysis).

##### Gel-free proteomics

Different fractions (TCL, LE/LYS and PM) isolated from WT and NPC1-KO HeLa cells were dissolved in 1 mL urea lysis buffer (20 mM HEPES pH 8.0, 8 M urea, 1 mM sodium orthovanadate, 2.5 mM sodium pyrophosphate and 1 mM β-glycerophosphate) at room temperature. Samples were sonicated (microtip at 20 W, 3 bursts of 3 s each, cooled on ice in between bursts) to ensure complete lysis and/or protein extraction, centrifuged (20,000 g, 15 min, 4 °C) and extracted proteins were reduced and alkylated using DTT (10 mM, 30 min at 55 °C) and IAA (20 mM, 15 min, 37 °C, dark). The protein concentration was measured by a Bradford assay and protein digestion was performed in two steps by first adding EndoLys-C (WAKO) in a 1:250 (w:w) ratio for 4 h at 37 °C after which the sample was diluted to 2 M urea and digested with trypsin (Promega) in a 1:100 (w:w) ratio for 16 h at 37 °C. The generated peptide mixture was cleaned-up, (C_18_ SPE cartridges - SampliQ, Agilent), vacuum dried and re-dissolved in loading solvent A (2% acetonitrile, 0.1% TFA) to a final concentration of 1 μg/μl.

Of each sample, 6 μl was injected for LC-MS/MS analysis on an Ultimate 3000 RSLC nano LC (Thermo Fisher Scientific, Bremen, Germany) in-line connected to a Q Exactive HF mass spectrometer (Thermo Fisher Scientific). Peptides were first loaded on a trapping column (made in-house, 100 μm internal diameter (I.D.) ×20 mm, 5 μm beads C18 Reprosil-HD, Dr. Maisch, Ammerbuch-Entringen, Germany). After flushing from the trapping column, peptides were loaded on an analytical column (made in-house, 75 μm I.D. ×400 mm, 3 μm beads C18 Reprosil-HD, Dr. Maisch) packed in the needle (pulled in-house). Peptides were loaded with loading solvent (0.1% TFA in water) and separated with a non-linear 150 min gradient of 2–56% solvent B (0.1% formic acid in water/acetonitrile, 20/80 (v/v)) at a constant flow rate of 250 nL/min. This was followed by a 10 min wash reaching 99% solvent B’ (0.1% formic acid in water/acetonitrile, 20/80 (v/v)) and re-equilibration with solvent A (0.1% FA in water). The column temperature was kept constant at 40 °C (CoControl 3.3.05, Sonation).

##### LC-MS/MS and data analysis

The mass spectrometer was operated in data-dependent, positive ionization mode, automatically switching between MS and MS/MS acquisition for the 16 most abundant peaks in a given MS spectrum. The source voltage was set to 3.0 kV and the capillary temperature was set to 250 °C. One MS1 scan (m/z 375–1500, AGC target 3E6 ions, maximum ion injection time of 80 ms) acquired at a resolution of 60,000 (at 200 m/z) was followed by up to 16 tandem MS scans at a resolution 15,000 (at 200 m/z) of the most intense ions fulfilling predefined selection criteria (AGC target 1E5 ions, maximum ion injection time of 60 ms, isolation window of 1.5 m/z, fixed first mass of 145 m/z, spectrum data type: centroid, underfill ratio 2%, intensity threshold 1.3E4, exclusion of unassigned, singly charged precursors, peptide match preferred, exclude isotopes on, dynamic exclusion time of 12 s). The HCD collision energy was set to 32% Normalized Collision Energy and the polydimethylcyclosiloxane background ion at 445.12002 Da was used for internal calibration (lock mass).

### Protein identification and quantification

#### Gel-based proteomics

From the MS/MS data in each LC-MS/MS run, Mascot Generic Files were created using the Mascot Distiller software (version 2.4.3.3, Matrix Science, www.matrixscience.com/Distiller). While generating these peak lists, grouping of spectra was allowed in Mascot Distiller with a maximal intermediate retention time of 30 s, and a maximum intermediate scan count of 5 was used where possible. Grouping was done with 0.005 Da precursor tolerance. A peak list was only generated when the MS/MS spectrum contained more than 10 peaks. There was no de-isotoping and the relative signal to noise limit was set at 2. These peak lists were then searched using the Mascot search engine (MatrixScience, www.matrixscience.com) using the Mascot Daemon interface (version 2.4, Matrix Science). Spectra were searched against the SwissProt restricted to Homo sapiens taxonomy. Variable modifications were set to methionine oxidation, pyro-glutamate formation of amino terminal glutamine, and acetylation of the protein N-terminus. Mass tolerance on precursor ions was set to 10 ppm (with Mascot’s C13 option set to 1), and on fragment ions to 20 mmu. The instrument setting was put on ESI-QUAD. Enzyme was set to trypsin, allowing for 1 missed cleavage. Only peptides that were ranked first and scored above the Mascot significance score threshold set at 99% confidence were withheld. The 99% threshold corresponds to a chance of 1 in 100 for an identified peptide to be a false positive (approximately corresponding to 1% FDR). The peptides were further mapped back to the original protein database using an in-house Perl script to filter out and keep only those peptides matching to one protein. Among all isoforms for a given protein, the representative isoform, being the one with the longest sequence, was considered.

#### Gel-free proteomics

Data analysis was performed with MaxQuant (version 1.5.3.8) using the Andromeda search engine with default search settings including a false discovery rate set at 1% on both the peptide and protein level. Spectra were searched against the human proteins in the UniProt/Swiss-Prot database (database release version of November 2015 containing 20,193 human protein sequences, www.uniprot.org). The mass tolerance for precursor and fragment ions was set to 4.5 and 20 ppm, respectively, during the main search. Enzyme specificity was set as C-terminal to arginine and lysine, also allowing cleavage at arginine/lysine-proline bonds with a maximum of two missed cleavages. Carbamidomethylation of cysteine residues was set as a fixed modification and variable modifications were set to oxidation of methionine residues (to sulfoxides) and acetylation of protein N-termini. Only proteins identified with at least one unique or razor peptide were withheld for further analysis.

Proteins were quantified by the MaxLFQ algorithm integrated in the MaxQuant software. A minimum ratio count of two unique or razor peptides was required for quantification. Further data analysis was performed with the Perseus software (version 1.5.2.4) after loading the protein groups file from MaxQuant. Potential contaminants, proteins identified only by site and reverse database hits were removed followed by the grouping of replicate samples. Proteins with less than two valid values in at least one group were removed and missing values were imputed from a normal distribution around the detection limit. Then, t-tests were further performed (FDR = 0.05 and S_0_ = 0.1) to compare KO and WT samples and to reveal significantly regulated proteins.

#### Proteomics database online

The mass spectrometry proteomics data have been deposited to the ProteomeXchange Consortium via the PRIDE partner repository with the dataset identifier PXD001225 (Gel-based proteomics) (Username: reviewer28919@ebi.ac.uk; Password: k0uc5Xgs) and PXD004273 (Gel-free proteomics) (Username: reviewer20693@ebi.ac.uk; Password: 93XyyPuw).

## Additional Information

**How to cite this article**: Tharkeshwar, A. K. *et al*. A novel approach to analyze lysosomal dysfunctions through subcellular proteomics and lipidomics: the case of NPC1 deficiency. *Sci. Rep.* **7**, 41408; doi: 10.1038/srep41408 (2017).

**Publisher's note:** Springer Nature remains neutral with regard to jurisdictional claims in published maps and institutional affiliations.

## Supplementary Material

Supplementary Information

Supplementary Table S1

Supplementary Table S2

Supplementary Table S3

Supplementary Table S4
