# Supplementary material for: A novel approach to analyze lysosomal dysfunctions through subcellular proteomics and lipidomics: the case of NPC1 deficiency
Source: Sci Rep. 2017 Jan 30;7:41408. doi: 10.1038/srep41408 (PMC5278418; doi:10.1038/srep41408)
Supplement: Supplementary Information [file srep41408-s1.pdf]

A novel approach to analyze lysosomal dysfunctions through subcellular proteomics and lipidomics: the case of NPC1 deficiency

Arun Kumar Tharkeshwar[1,2,3], Jesse Trekker[2,4], Wendy Vermeire[1], Jarne Pauwels[3], Ragna Sannerud[1], David A. Priestman[5], Danielle te Vruchte[5], Katlijn Vints[6], Pieter Baatsen[6], Jean-Paul Decuypere[1], Huiqi Lu[1], Shaun Martin[7], Peter Vangheluwe[7], Johannes V. Swinnen[8], Liesbet Lagae[2,9], Francis Impens[3,10], Frances M. Platt[5], Kris Gevaert[3], Wim Annaert[1*]

[1]Laboratory for Membrane Trafficking, VIB Center for the Biology of Disease, Center for Human Genetics (KU Leuven), Leuven, Belgium
[2]Department of Life Science Technology, imec, Leuven, Belgium
[3]VIB Medical Biotechnology Center & Department of Biochemistry, UGent, Ghent, Belgium
[4]Biomedical MRI, Department of Imaging and Pathology, KU Leuven, Leuven 3000, Belgium
[5]Department of Pharmacology, University of Oxford, Oxford, United Kingdom
[6]Bio-imaging core - Electron Microscopy Facility (EMCORF), VIB Center for the Biology of Disease, Leuven, Belgium
[7]Laboratory of Cellular Transport Systems, Department of Cellular and Molecular Medicine, KU Leuven, Leuven, Belgium
[8]Laboratory of Lipid Metabolism and Cancer, Department of Oncology, KU Leuven, Leuven, Belgium
[9]Department of Physics, Solid State Physics and Magnetism, KU Leuven, Leuven, Belgium
[10]Proteomics Expertise Center, VIB Medical Biotechnology Center, Ghent, Belgium

* Corresponding author:

Wim Annaert, PhD,

Laboratory for Membrane Trafficking, VIB & KU Leuven

Gasthuisberg, O&N4, Rm. 7.159, Herestraat 49, B-3000 Leuven, Belgium

Tel: +32 16 330520; Email: wim.annaert@cme.vib-kuleuven.be

Summary of Supplementary material

Supplementary Figure 1: Synthesis of monodisperse SPIONs using thermal decomposition followed by surface functionalization.

Supplementary Figure 2: Effect of functionalized SPIONs on Hela cell viability, internalization, endocytic transport and lysosomal function.

Supplementary Figure 3: Aminolipid-coated SPIONs accumulate at the cell surface while DMSA-coated SPIONs accumulate in Lamp1 positive organelles across various cell types.

Supplementary Figure 4: Fractions isolated using DMSA- and aminolipid-SPIONs are devoid of contaminating organelles.

Supplementary Figure 5: Proteomics on isolated subcellular compartments demonstrate enrichments for endosomal and cell surface associated GO-terms.

Supplementary Figure 6: KO of NPC1 causes increases in lysosomal volume and pH along with an increased storage of cholesterol and glycospingolipids.

Supplementary Figure 7: Lipids accumulating in LE/LYS of NPC1 KO cells do not show significant changes in their molecular features.

Supplementary Table S1: Absolute quantities of lipids identified in PM and LE/LYS fractions isolated from WT and NPC1 KO HeLa cells.

Supplementary Table S2: Individual features of the lipid species identified in PM and LE/LYS fractions isolated from WT and NPC1 KO HeLa cells.

Supplementary Table S3: List of proteins identified in LE/LYS fraction isolated from WT and NPC1 KO HeLa cells.

Supplementary Table S4: List of proteins identified in PM fraction isolated from WT and NPC1 KO HeLa cells.

Supplementary Figure Legends

Supplementary Figure 1: Synthesis of monodisperse SPIONs using The thermal decomposition method of SPION synthesis followed by its surface functionalization results in the formation of mono-dispersed particles with superparamagnetic property.(A) Scheme representing major steps in SPION synthesis. Surface functionalization has no influence on the superparamagnetic behavior **(B-G)** and size distribution **(H-N)** of SPIONs. Normalized magnetic hysteresis curves of SPIONs coated with oleic acid - DMSA **(B)**, oleic acid - lipids **(C)**, aminolipids **(D)**, DMSA **(E)**, carboxylipids **(F)**, methoxylipids **(G)** as measured by vibrating sample magnetometer (VSM). The red curve line represents the Langevin fitting. Histograms of the size distribution of SPIONs coated with oleic acid - DMSA **(H)**, oleic acid - lipids **(I)**, aminolipids **(J)**, DMSA **(K)**, carboxylipids **(L)**, methoxylipids **(M)** as determined by TEM were fitted with a log normal distribution curve (red). **(N)** FTIR spectral analysis of functionalized surface coatings. All FTIR-spectra, although less abundant for the DMSA-SPIONs, showed several similar peaks just below 3000 cm^{-1}. These peaks are characteristic for the asymmetric (ν_{as}) and symmetric (ν_s) stretch of saturated alkane chains (ν_{as} CH_3 ~2960 cm^{-1}, ν_{as} CH_2 ~2925 cm^{-1}, ν_s CH_3 ~2890 cm^{-1}, ν_s CH_2 ~2855 cm^{-1}), which are all present in the molecules functionalizing the SPIONs. A joint peak visible around ~600 cm^{-1} represents the Fe-O stretch of the ferrite core in the SPIONs while the presence of PEG in phospholipids is clearly visible due to the strong peak at ~1110 cm^{-1} which is derived from the ethylene oxide stretch (C-O-C). Due to the abundance of different FTIR vibrations in the phospholipids it is not possible to distinguish the specific end-groups (O-CH_3, -COOH, -NH_2) of different lipids used to functionalize the SPIONs.

Supplementary Figure 2: Effect of functionalized SPIONs on Hela cell viability, internalization, endocytic transport and lysosomal function. (A) Percentage of viable and dead cells after 1 h of incubation with functionalized SPIONs as measured with trypane blue. **(B-C)** SPIONs functionalized with either carboxylipids/methoxylipids do not interact with HeLa cells at 37°C. TEM images of HeLa cells incubated with carboxylipid- **(B)** or methoxylipid- **(C)** coated SPIONs

for 15 min and chased for 4 h. **(D)** XBP1 splicing assay and quantitative western blot analysis **(E-F)** of indicated marker proteins on total extracts of cells incubated with DMSA- and aminolipid-SPIONs for a chase period of 4 and 15 h. As a control, cells were incubated with thapsigargin. **(G)** Percentage of viable and dead (apoptotic) cells after a chase period of 4 h with aminolipid- and DMSA-SPIONs as measured with propidium iodide staining. **(H)** Western blot analysis for indicated marker proteins on total cell extracts of cells incubated with aminolipid- and DMSA-SPIONs for a chase period of 4 and 15 h. As a control, total cell extracts from cells incubated without SPIONs or with Bortezomib were used. **(I)** Cell surface biotinylation on HeLa cells incubated (pulse 15 min, chase 4 h at 37°C) with and without DMSA-SPIONs using EZ-Link Sulfo-NHS-SS-Biotin (Pierce) (10 min at 4 °C). Representative Western blot of biotinylated transferrin receptor (TfR) at indicated internalization time points at 37°C. **(L)** Confocal analysis of Alexa568-labeled transferrin internalized for 20min at 37 °C. Insets show zoomed areas depicting colocalizaiton with endogenous EEA1. **(M)** Quantification of co-localization represented by the Pearson's coefficient. **(J)** DQ-BSA assay measuring lysosomal proteolytic activity in HeLa cells incubated with DMSA-coated SPIONs for 15 min and chased for 4 and 15 h. As a control, total cell extracts from cells were incubated without SPIONs or with bafilomycin A1. Confocal image of LE/LYS isolates stained with LysoTracker-red **(K)**. Ctrl - Control, TG - Thapsigargin, BR - Bortezomib, BAF - bafilomycin A1, Amino - Aminolipid-SPIONs, DMSA - DMSA-SPIONs, Carb - Carboxylipid-SPIONs and Meth - Methoxylipid-SPIONs, NR - Nonreduced, TfR - Transferrin receptor. Scale bar = 20 µm.

Supplementary Figure 3: Aminolipid-coated SPIONs accumulate at the cell surface while DMSA-coated SPIONs accumulate in Lamp1 positive organelles across various cell types. Confocal analysis of MEF, HeLa, MF4, RAW and rat primary hippocampal neurons incubated with fluorescently modified DMSA-coated SPIONs (pulse 15 min, chase 4 h, 37°C) **(A)** or with **(B)** aminolipid-coated SPIONs (pulse 15 min, 4°C). Scale bar in **(A)** is 10 µm, **(B)** is 20 µm.

Supplementary Figure 4: Fractions isolated using DMSA- and aminolipid-SPIONs are devoid of contaminating organelles. Quantitative western blot analysis of the indicated marker proteins in isolated fractions of DMSA-SPIONs with increasing chase periods of **(A)** 0 min **(B)** 1 h **(C)** 2 h **(D)** 3 h **(E)** 4 h and in isolated fractions of aminolipid-SPIONs **(F)** as a fold increase relative to the total cell lysate (TCL) (mean ± SEM, n=3). Rab5 and CatD are early and LE/LYS marker proteins, respectively; p58 is used as a marker for intermediate to cis-Golgi compartments, lamin as a nuclear marker, actin as a cytoskeletal marker and PEX14p is a peroxisomal marker protein.

Supplementary Figure 5: Proteomics on isolated subcellular compartments demonstrate enrichments for endosomal and cell surface associated GO-terms. Venn diagram indicating the distribution of unique proteins identified in two replicates using gel-based shotgun proteome analysis of the total cell lysate (TCL) **(A1)** and magnetically isolated fractions using aminolipid-coated **(A2)**, DMSA-coated (chase period of 4 h) SPIONs **(A3)**. Gene Ontology (GO) enrichment analysis of proteins identified in the bound/magnetic fractions isolated using DMSA- (B1, B2), or aminolipid-SPIONs (C1, C2). GO enrichment was performed using DAVID.

Supplementary Figure 6: KO of NPC1 causes increases in lysosomal volume and pH along with an increased storage of cholesterol and glycospingolipids. (A) Western blot analysis of the indicated organelle marker proteins in total cell lysates shows complete loss of NPC1 protein expression in the two KO cell lines. Rab5 is used as a marker for early endosomes, Rab7 for late endosomes, Lamp1 for lysosomes, ribophorin, actin and GAPDH represent endoplasmic reticulum, cytoskeleton and cytosol respectively. Relative LE/LYS volume and pH increases in NPC1-KO cells **(B-C)**. Fluorescence of cells stained with LysoTracker-green **(B)** or LysoSensor Yellow/Blue DND-160 **(C)** was measured by flow cytometry and corresponding values are expressed as mean equivalent of fluorescence (MEFL). **(D)** Filipin staining reveals cholesterol accumulation in LAMP1-positive organelles solely in NPC1 KO cells. NPC1-KO cells show relative increase in the storage of sphingosine **(E)** and glycospingolipid **(F)** as measured by HPLC.

Supplementary Figure 7: Lipids accumulating in LE/LYS of NPC1 KO cells do not show significant changes in their molecular features. Enrichment plot for Glycerophospholipid (GPL) and Sphingolipid (SP) chain lengths expressed as number of carbon atoms **(A, D and H, K)**, saturation expressed as number of double bonds **(B, E and I, L)** related to the sum of long chain fatty acid moieties, and the number of hydroxylations **(C, F and J, M)** and this for PM and LE/LYS fractions isolated from NPC1 KO cells relative to WT HeLa cells. Features were calculated from individual quantities of lipid molecular species (mean ± SEM, n=3).

Supplementary Table Legends:

Supplementary Table S1: Absolute quantities of lipids identified in PM and LE/LYS fractions isolated from WT and NPC1 KO HeLa cells. Values are expressed in pmol for each lipid species identified in each individual sample, experiment.

Supplementary Table S2: Individual features of the lipid species identified in PM and LE/LYS fractions isolated from WT and NPC1 KO HeLa cells. Features are normalized to individual lipid class.

Supplementary Table S3: List of proteins identified in LE/LYS fraction isolated from WT and NPC1 KO HeLa cells.

Supplementary Table S4: List of proteins identified in PM fraction isolated from WT and NPC1 KO HeLa cells.

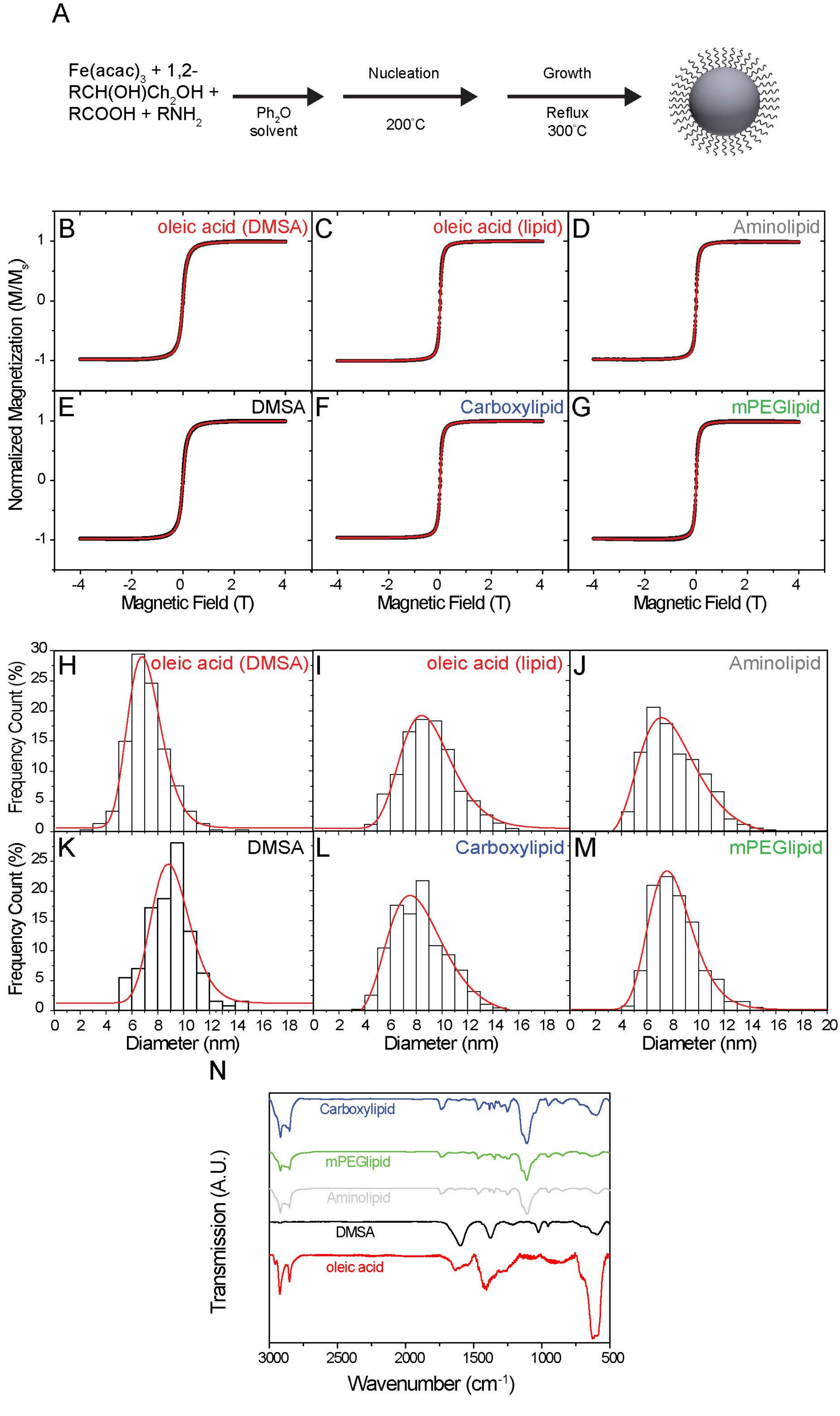
A
Fe(acac)3 + 1,2-RCH(OH)Ch2OH + RCOOH + RNH2
Ph2O solvent
Nucleation
200°C
Growth
Reflux 300°C
B oleic acid (DMSA)
C oleic acid (lipid)
D Aminolipid
E DMSA
F Carboxylipid
G mPEGlipid
Normalized Magnetization (M/Ms)
Magnetic Field (T)
H oleic acid (DMSA)
I oleic acid (lipid)
J Aminolipid
K DMSA
L Carboxylipid
M mPEGlipid
Frequency Count (%)
Diameter (nm)
N
Carboxylipid
mPEGlipid
Aminolipid
DMSA
oleic acid
Transmission (A.U.)
Wavenumber (cm-1)

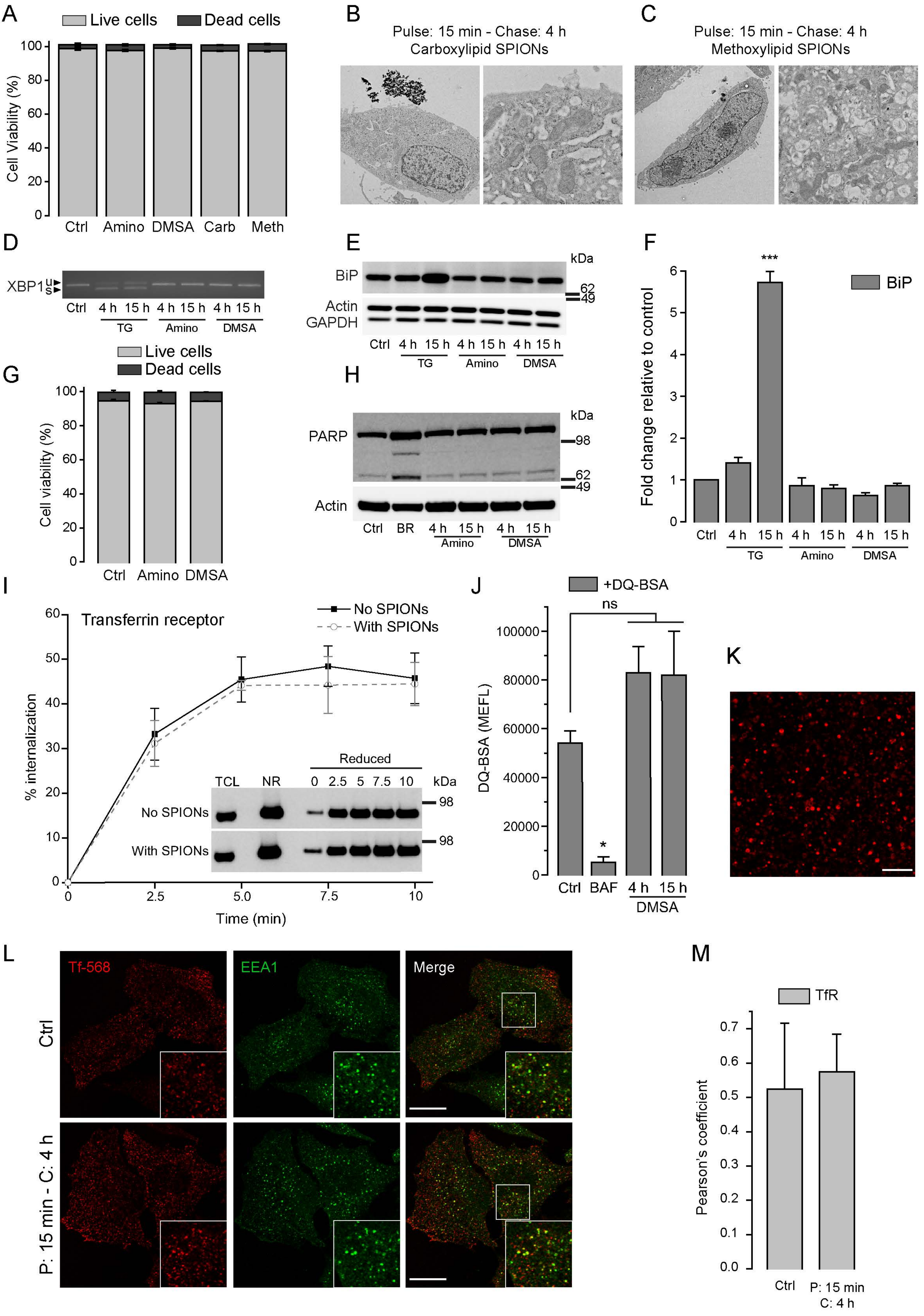

A
Live cells
Dead cells
Cell Viability (%)
Ctrl Amino DMSA Carb Meth
B
Pulse: 15 min - Chase: 4 h
Carboxylipid SPIONs
C
Pulse: 15 min - Chase: 4 h
Methoxylipid SPIONs
D
XBP1
u
s
Ctrl 4 h 15 h 4 h 15 h 4 h 15 h
TG Amino DMSA
E
BiP
Actin
GAPDH
kDa
62
49
Ctrl 4 h 15 h 4 h 15 h 4 h 15 h
TG Amino DMSA
F
***
BiP
Fold change relative to control
Ctrl 4 h 15 h 4 h 15 h 4 h 15 h
TG Amino DMSA
G
Live cells
Dead cells
Cell viability (%)
Ctrl Amino DMSA
H
PARP
Actin
kDa
98
62
49
Ctrl BR 4 h 15 h 4 h 15 h
Amino DMSA
I
Transferrin receptor
No SPIONs
With SPIONs
% internalization
Time (min)
Reduced
TCL NR 0 2.5 5 7.5 10 kDa
No SPIONs
With SPIONs
98
J
+DQ-BSA
ns
DQ-BSA (MEFL)
*
Ctrl BAF 4 h 15 h
DMSA
K
L
Tf-568
EEA1
Merge
Ctrl
P: 15 min - C: 4 h
M
TfR
Pearson's coefficient
Ctrl
P: 15 min
C: 4 h

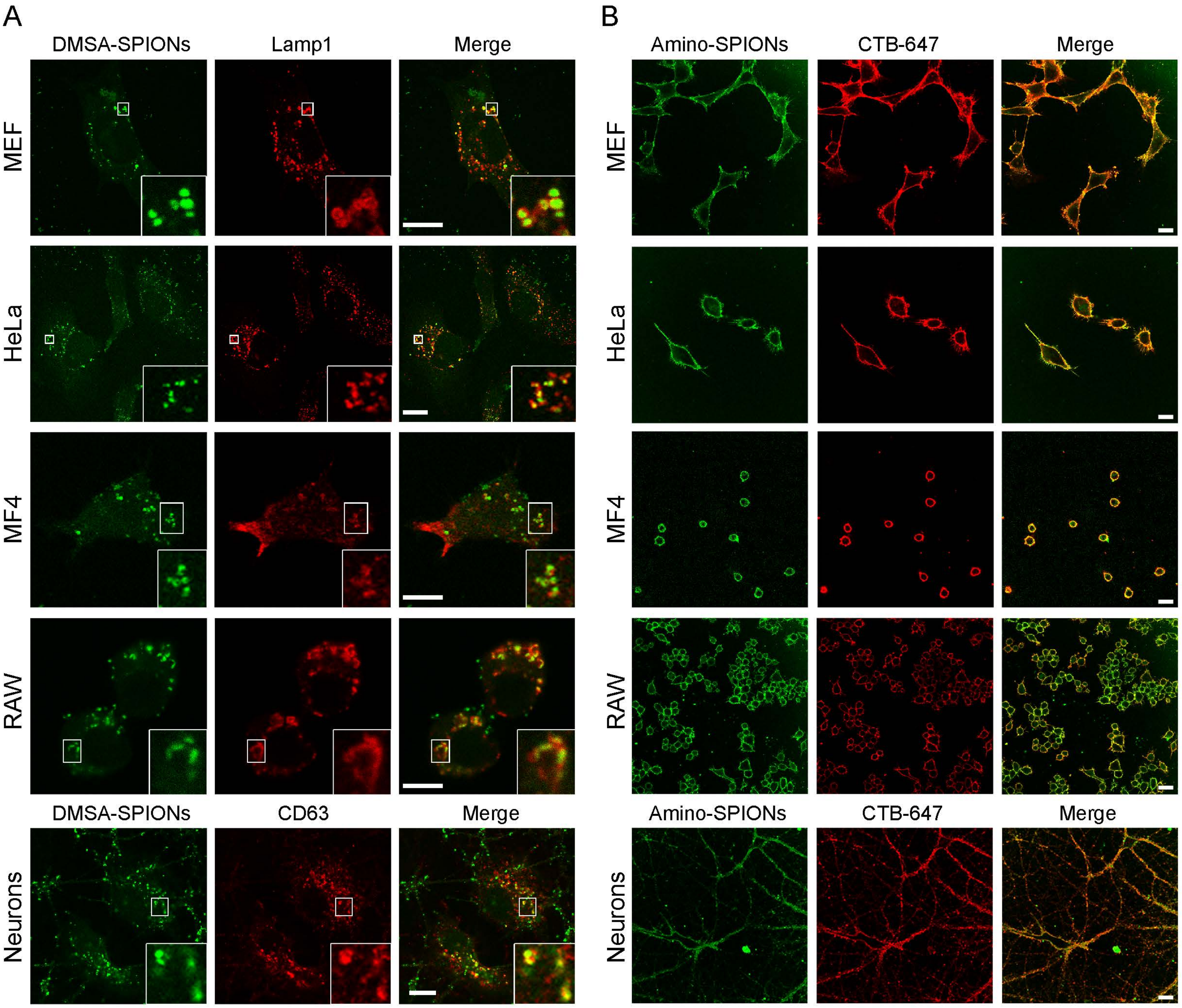
A
DMSA-SPIONs
Lamp1
Merge
MEF
HeLa
MF4
RAW
DMSA-SPIONs
CD63
Merge
Neurons
B
Amino-SPIONs
CTB-647
Merge
MEF
HeLa
MF4
RAW
Amino-SPIONs
CTB-647
Merge
Neurons

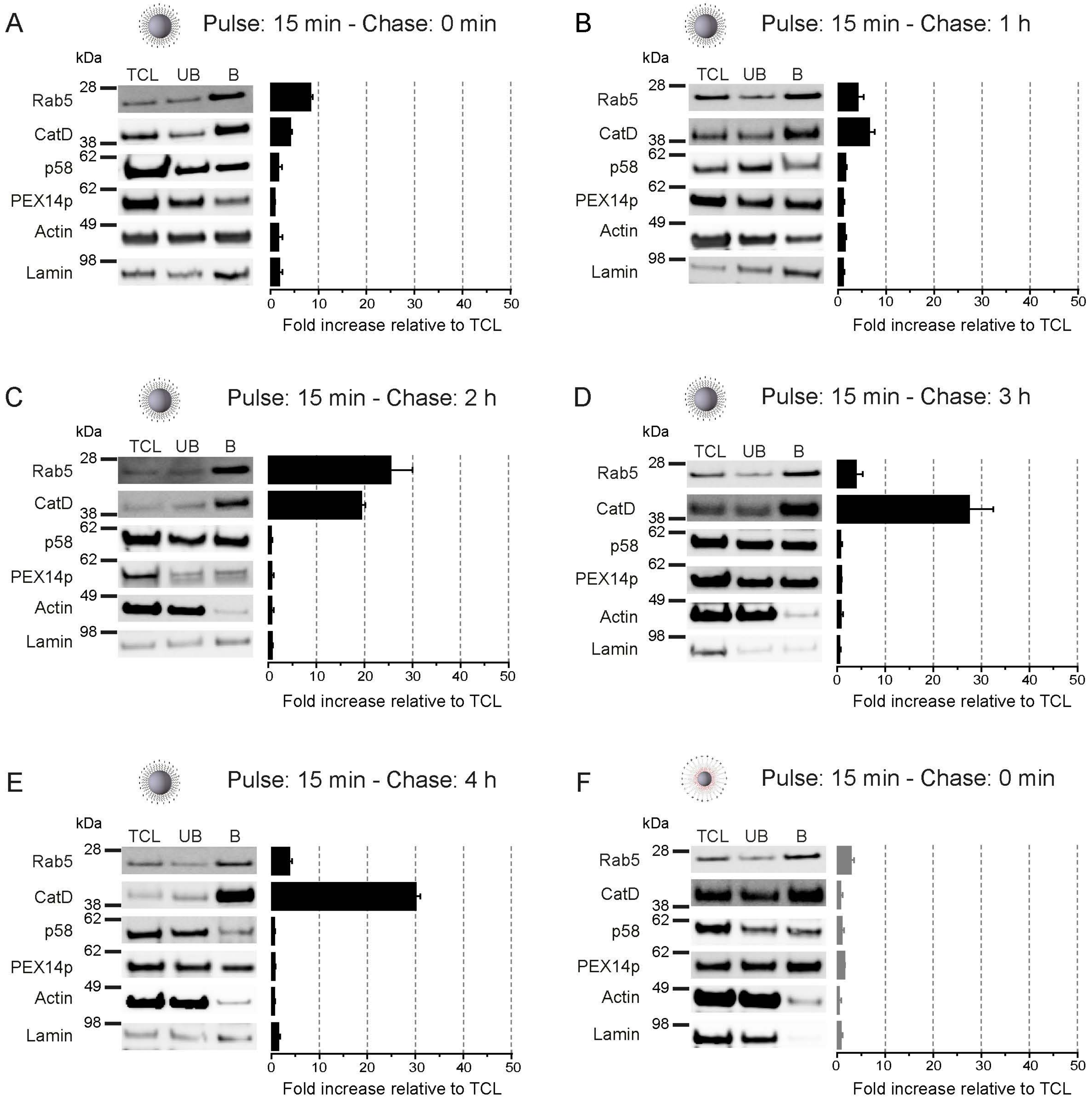

A
Pulse: 15 min - Chase: 0 min
kDa
TCL UB B
28
Rab5
CatD
38
62
p58
62
PEX14p
49
Actin
98
Lamin
0 10 20 30 40 50
Fold increase relative to TCL
B
Pulse: 15 min - Chase: 1 h
kDa
TCL UB B
28
Rab5
CatD
38
62
p58
62
PEX14p
49
Actin
98
Lamin
0 10 20 30 40 50
Fold increase relative to TCL
C
Pulse: 15 min - Chase: 2 h
kDa
TCL UB B
28
Rab5
CatD
38
62
p58
62
PEX14p
49
Actin
98
Lamin
0 10 20 30 40 50
Fold increase relative to TCL
D
Pulse: 15 min - Chase: 3 h
kDa
TCL UB B
28
Rab5
CatD
38
62
p58
62
PEX14p
49
Actin
98
Lamin
0 10 20 30 40 50
Fold increase relative to TCL
E
Pulse: 15 min - Chase: 4 h
kDa
TCL UB B
28
Rab5
CatD
38
62
p58
62
PEX14p
49
Actin
98
Lamin
0 10 20 30 40 50
Fold increase relative to TCL
F
Pulse: 15 min - Chase: 0 min
kDa
TCL UB B
28
Rab5
CatD
38
62
p58
62
PEX14p
49
Actin
98
Lamin
0 10 20 30 40 50
Fold increase relative to TCL

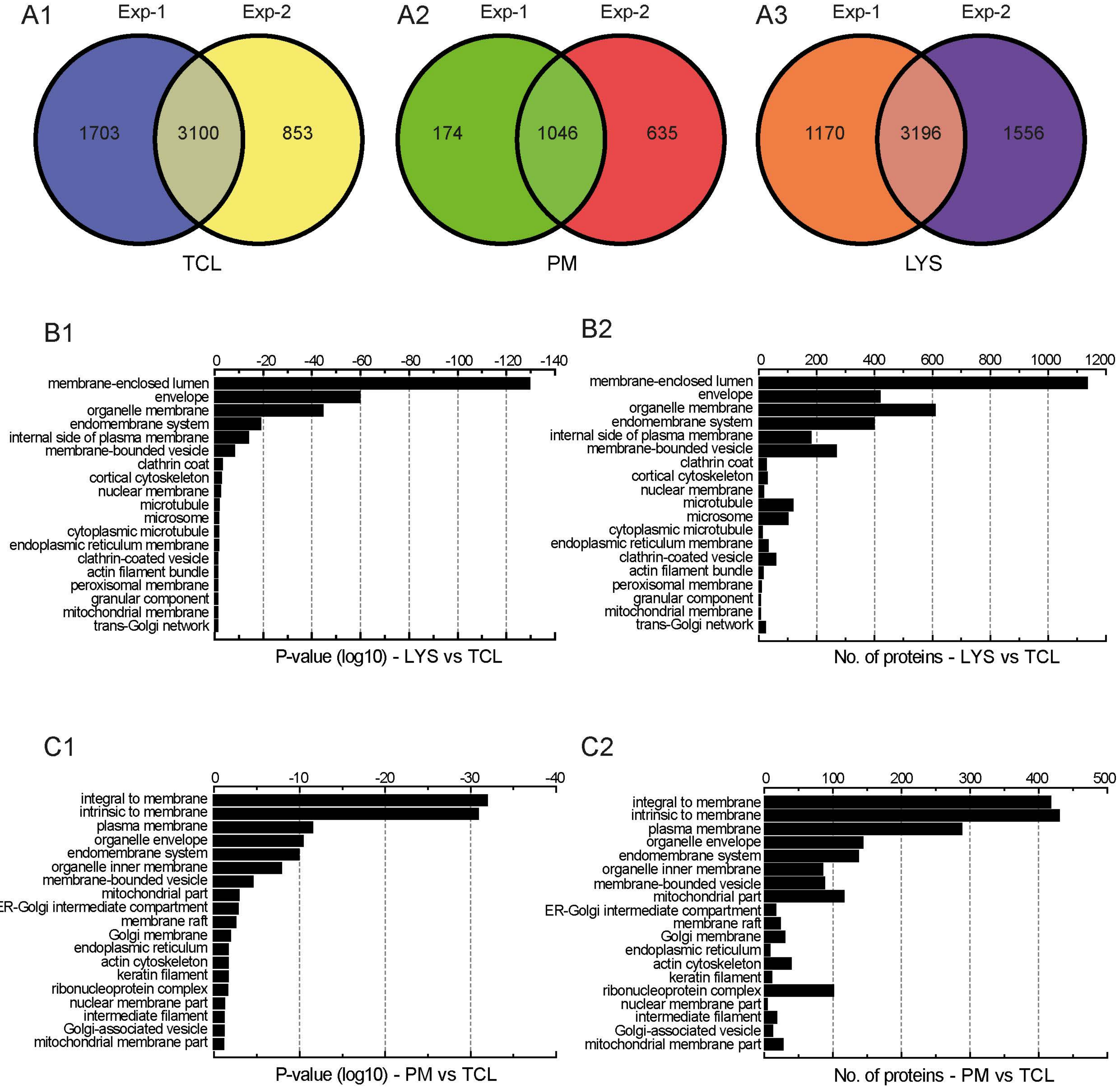
A1
Exp-1
Exp-2
1703
3100
853
TCL
A2
Exp-1
Exp-2
174
1046
635
PM
A3
Exp-1
Exp-2
1170
3196
1556
LYS
B1
0 -20 -40 -60 -80 -100 -120 -140
membrane-enclosed lumen
envelope
organelle membrane
endomembrane system
internal side of plasma membrane
membrane-bounded vesicle
clathrin coat
cortical cytoskeleton
nuclear membrane
microtubule
microsome
cytoplasmic microtubule
endoplasmic reticulum membrane
clathrin-coated vesicle
actin filament bundle
peroxisomal membrane
granular component
mitochondrial membrane
trans-Golgi network
P-value (log10) - LYS vs TCL
B2
0 200 400 600 800 1000 1200
No. of proteins - LYS vs TCL
C1
0 -10 -20 -30 -40
integral to membrane
intrinsic to membrane
plasma membrane
organelle envelope
endomembrane system
organelle inner membrane
membrane-bounded vesicle
mitochondrial part
ER-Golgi intermediate compartment
membrane raft
Golgi membrane
endoplasmic reticulum
actin cytoskeleton
keratin filament
ribonucleoprotein complex
nuclear membrane part
intermediate filament
Golgi-associated vesicle
mitochondrial membrane part
P-value (log10) - PM vs TCL
C2
0 100 200 300 400 500
No. of proteins - PM vs TCL

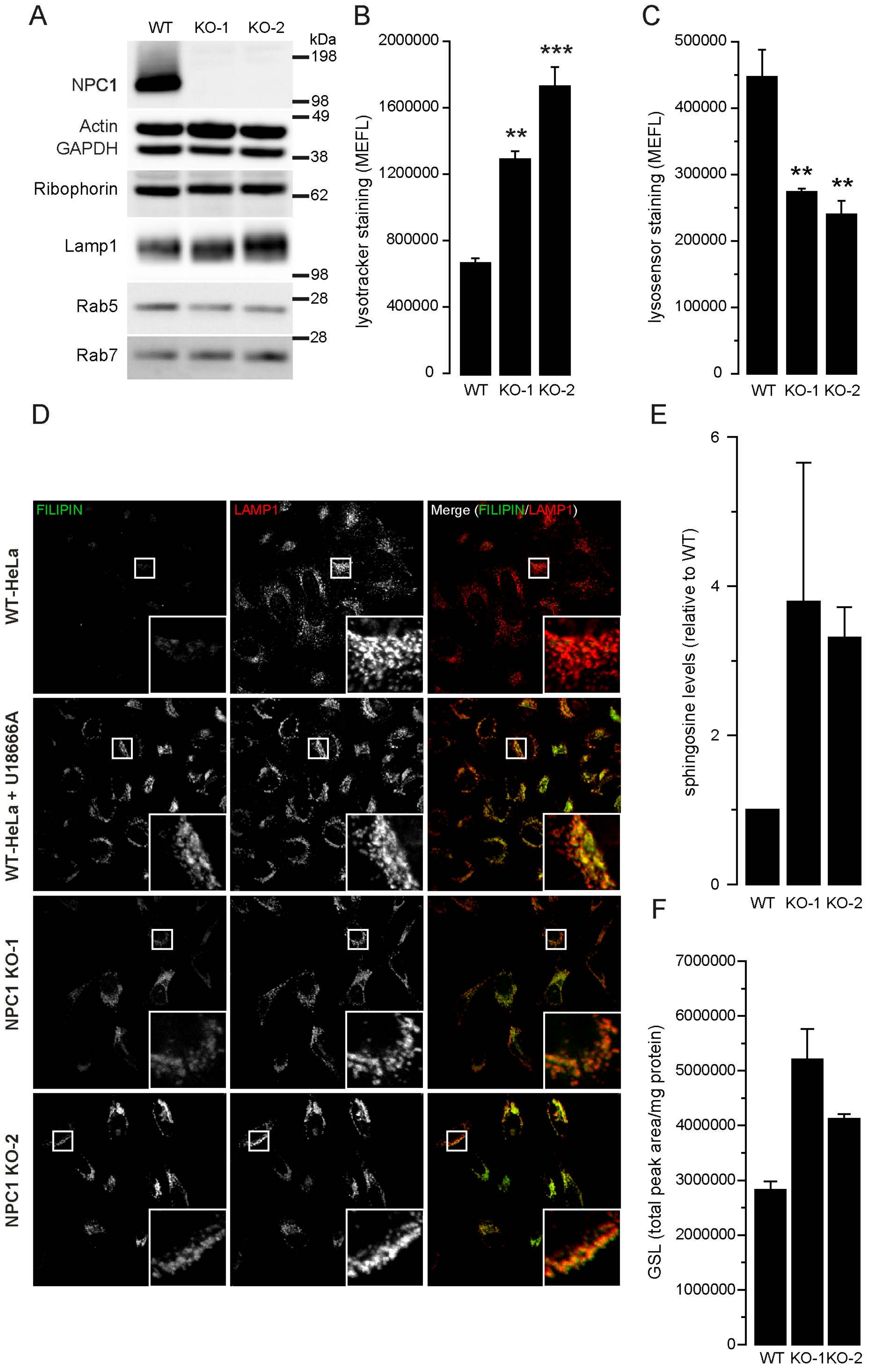

A
WT KO-1 KO-2
kDa
198
NPC1
98
49
Actin
GAPDH
38
Ribophorin
62
Lamp1
98
28
Rab5
28
Rab7
B
2000000
1600000
1200000
800000
400000
0
lysotracker staining (MEFL)
**
***
WT KO-1 KO-2
C
500000
400000
300000
200000
100000
0
lysosensor staining (MEFL)
**
**
WT KO-1 KO-2
D
FILIPIN
LAMP1
Merge (FILIPIN/LAMP1)
WT-HeLa
WT-HeLa + U18666A
NPC1 KO-1
NPC1 KO-2
E
6
4
2
0
sphingosine levels (relative to WT)
WT KO-1 KO-2
F
7000000
6000000
5000000
4000000
3000000
2000000
1000000
0
GSL (total peak area/mg protein)
WT KO-1KO-2

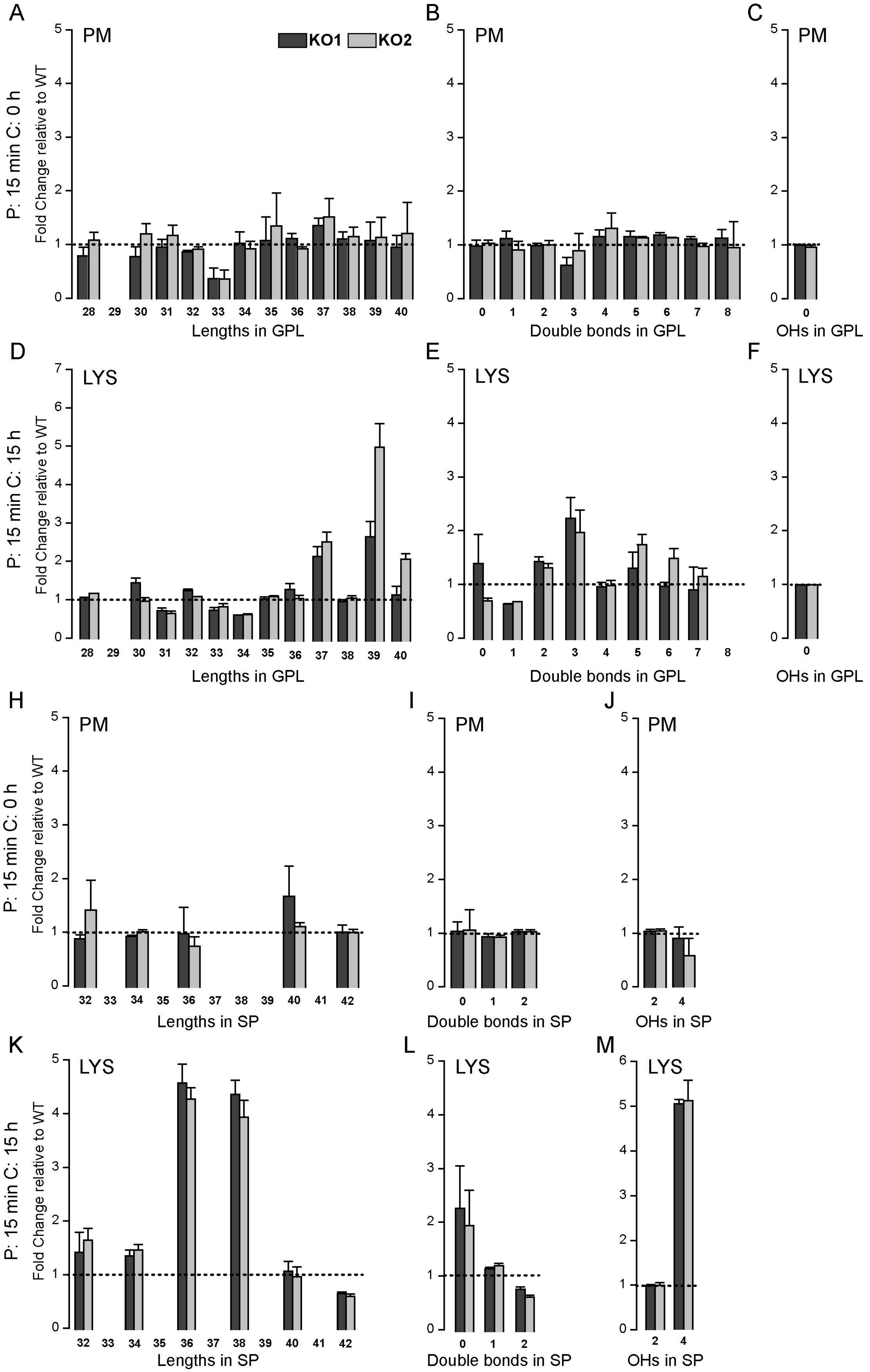

A
B
C
D
E
F
H
I
J
K
L
M
PM
LYS
KO1
KO2
P: 15 min C: 0 h
P: 15 min C: 15 h
Fold Change relative to WT
Lengths in GPL
Double bonds in GPL
OHs in GPL
Lengths in SP
Double bonds in SP
OHs in SP
